# Supplementary material for: Geography and availability of natural habitat determine whether cropland intensification or expansion is more detrimental to biodiversity
Source: Nat Ecol Evol. 2025 May 1;9(6):993–1008. doi: 10.1038/s41559-025-02691-x (PMC12148938; doi:10.1038/s41559-025-02691-x)

nature ecology & evolution

Supplementary information

https://doi.org/10.1038/s41559-025-02691-x

Geography and availability of natural habitat determine whether cropland intensification or expansion is more detrimental to biodiversity

In the format provided by the authors and unedited

Table of contents

Supplementary methods 2
Further details on the model explanatory variables 2
EarthStat 2
MapSPAM 2
Percentage of natural habitat 3
Subsistence yield as proxy for agricultural suitability 4
Statistical analysis 4
Supplementary tables 5
Data distribution 5
Non-crop 5
Maize 6
Soy 6
Wheat 7
Rice 7
Tables summarising information across all models 8
Model comparisons 12
Species Richness 13
Abundance 16
Relative abundance-weighted average range-size (RCAR) 20
Poisson and negative binomial estimate comparison 23
Frequentist and Bayesian estimate comparison 25
Supplementary figures 31
Data points distribution 31
Model checks 33
Species richness – Poisson models 33
Species richness – Negative binomial models 38
Total abundance 44
Relative abundance-weighted average range-size (RCAR) 49
Crop and yield gaps spatial data 56
Tests of assumptions of the expansion and intensification scenarios 60

Supplementary methods

Further details on the model explanatory variables

EarthStat

The EarthStat authors preferentially collected subnational agricultural statistics at one (state or province) or two administrative levels (county or district) below the national level, where available[1,2]. Consequently, data were collected from 2,299 administrative units one level below national from 150 countries (72.8% of the total) and 19,751 units two levels below national from 73 countries (35.4% of the total), with data sometimes available at different subnational levels for different areas of the same country. A single value for the lowest available administrative unit was calculated by averaging all the information available for the years between 1997 and 2003. The average reported statistics were then disaggregated spatially onto a gridded map according to the fraction of each cell covered by cropland. Further information regarding the methodology for generating the EarthStat estimates can be found in Monfreda *et al.*[1] and Ramankutty *et al.*[2].

For EarthStat, the largest data source of subnational agricultural statistics was Agro-MAPS[3]. Other data sources were national census agencies and agricultural surveys for the largest countries such as United States, Brazil and China[1]. In the absence of subnational statistics, Monfreda *et al.*[1] used national-level data from the Food and Agricultural Organisation[4], and independent national level data from Afghanistan, Iraq, Somalia, and Taiwan that were not present in the FAO database. EarthStat data include spatial layers on yield in tons per hectare, data quality for yield information, total production in tons, fractional harvested area, harvested area in hectares, and data quality for harvested area for each crop in each grid cell. We have use in the current analysis the spatial layers on yield in tons per hectare, data quality for yield information, and harvested area in hectares.

MapSPAM

MapSPAM agricultural estimates were created with the Spatial Production Allocation Model (SPAM), a model produced by the International Food Policy Research Institute[5]. Similarly to EarthStat data, MapSPAM relies on sub-national reported statistics sourced from Agro-MAPS and on additional data collected by authors through research partnerships. MapSPAM prioritises sub-national reported statistics complemented with data at coarser resolutions but, as opposed to EarthStat, it does not include spatial information on the resolution of the underlying agricultural data. Therefore, it was not possible to select spatial estimates based on the highest resolutions. The sub-national crop data of MapSPAM were collected from 2,758 administrative units one level below national

(compared to 2,299 for EarthStat) and 21,498 units two levels below national (compared to 19,751 for EarthStat). Where in disagreement, the sub-national data were calibrated to agree with the national FAO data[6]. For the rest of the area, FAO national statistics were used. The reported statistics used for each time step were averaged over the three years around the reference year (for example, 1999-2001 for year 2000)[5].

SPAM uses a range of variables (e.g., probability of crop existence in a spatial unit, biophysical potential, area constraints, management constraints) to guide the spatial allocation of agricultural data into a global 5' x 5' (~10 km x 10 km at equator) gridded representation of cultivated area, production quantity and yield under different management regimes for the years 2000, 2005 and 2010[7].

For the year 2000, MapSPAM estimates were available for 20 crops, while all other crops were included in an additional catch-all group. For the years 2005 and 2010, the estimates include 42 crop categories including the catch-all group.

Through a combination of data and expert opinion, MapSPAM authors further disaggregate agricultural estimates into four levels according to farming technology and crop management systems: irrigated, rainfed high inputs, rainfed low inputs and rainfed subsistence. For a few large countries, management information was collected at the level of the first subnational administrative level below national[7]. For the rest of the countries, the authors collected information at national level on the ratios of yields under irrigated and rainfed conditions, and under high-input rainfed and low-input rainfed conditions for each crop. They then use these two ratios and areas under different production systems to calculate estimates of yield under different management systems, using reported yields as average yields across all the production systems within a pixel. We used MapSPAM subsistence estimates, which assumes rainfed conditions and no inputs, to check for the robustness of models to choice of subsistence yield.

Percentage of natural habitat

The original dataset at 30-arc-second resolution from Hoskins et al[8] includes estimates of proportional cover for five land-use classes (urban, cropland, pasture, secondary habitat and primary habitat). These estimates were obtained by downscaling the Land-use Harmonization dataset[9], a global time series of land use at a resolution of 0.5° that covers the time period 1500-2100. Hoskins *et al.*[8] downscale estimates for 2005, based on statistical

relationships with fine-scale data on climate, land cover, landform, human population density and accessibility defined at the level of 61 unique combinations of biomes and biogeographical realms.

Subsistence yield as proxy for agricultural suitability

We used subsistence yields modelled by a version of the Environmental Policy Integrated Climate (EPIC) model, which was initially developed in the United States to model how soil productivity is affected by agricultural management[10]. Since then, it has been expanded to allow the simulation of yields and agricultural processes at global and regional scales, and their interactions with agricultural management[11–13]. EPIC uses a daily time step and includes components on crop growth, weather simulation, competition, water, carbon and nutrient cycling, and soil processes. Simulated potential plant growth depends on conversion of CO_2 to biomass, intercepted solar radiation and vapour pressure deficit, while stresses from temperature, nutrient deficit, salinity and other soil factors lead to decreased plant growth.

We used yield simulated with EPIC-BOKU under a subsistence management system without fertilisation or irrigation at 0.5° x 0.5° resolution. The simulation used the WFDEI meteorological forcing data, which represents historical daily-resolution climate data on land at 0.5° × 0.5° resolution based on ERA-Interim reanalysis data[14]. For MapSPAM models, we averaged EPIC-BOKU subsistence yield estimates between the years 1997-2003 for the 2000 yields, 2002-2008 for the 2005 yields, and 2007-2013 for the 2010 yields.

Statistical analysis

We tested for correlations among all explanatory variables for both the yield-biodiversity and land-conversion models. Collinearity among variables was generally low with most correlations between variables being very weak (0 to 0.2) or weak (0.2 to 0.4). A few pairs of variables were moderately correlated (0.4 to 0.6) and a maximum of one pair of variables were strongly correlated (0.6 to 0.8) for some of our statistical models (Supplementary Table 6). When a pair of variables was strongly correlated, we eliminated one of them (Supplementary Table 6), prioritising yield, percentage of natural habitat and subsistence yield measures (in this order) to be kept for modelling. In all cases, it was climate variables (annual mean temperature and annual precipitation) that had to be removed due to high correlations with EarthStat yield, Epic-BOKU subsistence yield or, in the case of the yield-biodiversity model for rice, with percentage natural habitat.

Supplementary tables

Data distribution

Non-crop

Supplementary Table 1. Distribution of sites in the PREDICTS database used for the land-conversion models across land-use types and tropical/non-tropical regions. The acronyms used in the header are: SR – species richness and RCAR - relative abundance-weighted community-average range size.

Geographic region	Land use	Landscape	SR data points	Abundance data points	RCAR data points
non-tropical	Cropland	Human-modified landscape	1,202	1,032	848
		Natural landscape	69	68	39
	Primary vegetation	Human-modified landscape	579	516	274
		Natural landscape	1,389	1,199	488
tropical	Cropland	Human-modified landscape	167	91	58
		Natural landscape	194	174	81
	Primary vegetation	Human-modified landscape	203	107	32
		Natural landscape	1,525	1,314	673

Maize

Supplementary Table 2. Distribution of sites in the PREDICTS database in areas used to grow maize across land-use types and tropical/non-tropical regions. The acronyms used in the header are: SR – species richness and RCAR - relative abundance-weighted community-average range size.

Geographic region	Land use	EarthStat SR data points	EarthStat Abundance data points	EarthStat RCAR data points	MapSPAM SR data points	MapSPAM Abundance data points	MapSPAM RCAR data points
non-tropical	Cropland	1,589	1,411	1,182	1,269	1,091	919
	Primary vegetation	1,419	1,001	566	1,065	684	468
tropical	Cropland	518	417	296	395	321	245
	Primary vegetation	1,336	1,020	645	968	774	462

Soy

Supplementary Table 3. Distribution of sites in the PREDICTS database in areas used to grow soybean across land-use types and tropical/non-tropical regions. The acronyms used in the header are: SR – species richness and RCAR - relative abundance-weighted community-average range size.

Geographic region	Land use	EarthStat SR data points	EarthStat Abundance data points	EarthStat RCAR data points	MapSPAM SR data points	MapSPAM Abundance data points	MapSPAM RCAR data points
non-tropical	Cropland	885	711	579	736	562	450
	Primary vegetation	714	596	510	511	410	377
tropical	Cropland	279	227	158	178	161	130
	Primary vegetation	526	336	236	247	198	109

Wheat

Supplementary Table 4. Distribution of sites in the PREDICTS database in areas used to grow wheat across land-use types and tropical/non-tropical regions. The acronyms used in the header are: SR – species richness and RCAR - relative abundance-weighted community-average range size.

Geographic region	Land use	EarthStat SR data points	EarthStat Abundance data points	EarthStat RCAR data points	MapSPAM SR data points	MapSPAM Abundance data points	MapSPAM RCAR data points
non-tropical	Cropland	1,695	1,517	1,243	1,681	1,503	1,240
	Primary vegetation	1,319	921	551	1,131	744	464
tropical	Cropland	81	78	68	81	78	68
	Primary vegetation	132	89	78	85	65	65

Rice

Supplementary Table 5. Distribution of sites in the PREDICTS database in areas used to grow rice across land-use types and tropical/non-tropical regions. The acronyms used in the header are: SR – species richness and RCAR - relative abundance-weighted community-average range size.

Geographic region	Land use	EarthStat SR data points	EarthStat Abundance data points	EarthStat RCAR data points	MapSPAM SR data points	MapSPAM Abundance data points	MapSPAM RCAR data points
non-tropical	Cropland	319	315	232	243	239	163
	Primary vegetation	418	274	217	229	118	113
tropical	Cropland	525	423	308	415	343	269
	Primary vegetation	1,548	1,189	632	1,103	876	402

Tables summarising information across all models

Supplementary Table 6. Summary of the variables removed from the model selection process due to correlation. When a pair of variables had an absolute correlation value higher than 0.6, we eliminated one of them, prioritising yield, percentage of natural habitat and subsistence yield measures (in this order) to be kept for modelling

Response variable	Crop	Correlated variables removed from models	Correlation value
Species richness	Non-crop	-	-
	Maize	Annual mean temperature (correlated with EarthStat yield)	-0.68
	Soy	-	-
	Wheat	-	-
	Rice	Annual precipitation (correlated with % natural habitat)	0.8
Total abundance	Non-crop	-	-
	Maize	Annual mean temperature (correlated with EarthStat yield)	-0.7
		Annual precipitation (correlated with EarthStat yield)	-0.64
	Soy	-	-
	Wheat	-	-
	Rice	Annual precipitation (correlated with % natural habitat)	0.78
RCAR	Non-crop	-	-
	Maize	Annual mean temperature (correlated with EarthStat yield)	-0.73
		Annual precipitation (correlated with EarthStat yield)	-0.7
	Soy	Annual precipitation (correlation with EPIC-BOKU subsistence yield for the year 2000)	0.62
	Wheat	Annual precipitation (correlation with EPIC-BOKU subsistence yield for the year 2000)	0.64
	Rice	Annual precipitation (correlated with % natural habitat)	0.78

Supplementary Table 7. R formulae of best selected models. C represents a constant that is used in the transformation of certain variables (see the *Model Explanatory Variables* subsection in Methods for more details).

Response variable	Crop	Formula
Species richness	Conversion model	Species richness ~ LU-LS categories + GeographicRegion + annual mean temperature + log(annual precipitation) + LU-LS categories: log(C - duration human modification) + log(C - duration human modification) + (1|SS) + (1|SSB) + (1|SSBS)
	Maize	Species richness ~ LandUse + GeographicRegion + EarthStat yield + EarthStat yield 10 km buffer-focal difference + Crop Diversity + % natural habitat + % natural habitat:LandUse + % natural habitat:GeographicRegion + (1|SS) + (1|SSB) + (1|SSBS)
	Soy	Species richness ~ % natural habitat + log(annual precipitation) + % natural habitat:LandUse + LandUse + (1|SS) + (1|SSB) + (1|SSBS)
	Wheat	Species richness ~ GeographicRegion + EarthStat yield + EarthStat yield 10 km buffer-focal difference + % natural habitat + annual mean temperature + log(annual precipitation) + EarthStat yield:% natural habitat + EarthStat yield:LandUse + % natural habitat:GeographicRegion + % natural habitat:LandUse + LandUse + (1|SS) + (1|SSB) + (1|SSBS)
	Rice	Species richness ~ LandUse + EPIC BOKU subsistence yield + log(EarthStat yield):% natural habitat + % natural habitat:LandUse + log(EarthStat yield) + % natural habitat + (1|SS) + (1|SSB) + (1|SSBS)
Total abundance	Conversion model	Log(total abundance) ~ LU-LS categories + GeographicRegion + annual mean temperature + LU-LS categories:GeographicRegion + (1|SS) + (1|SSB)
	Maize	Log(total abundance) ~ LandUse + GeographicRegion + EarthStat yield + EarthStat 10-km-buffer yield difference + CropDiversity + log(C - duration human modification) + EarthStat yield:EPIC BOKU subsistence yield + EarthStat yield:% natural habitat + EarthStat yield:LandUse + EarthStat yield:GeographicRegion + EPIC BOKU subsistence yield + % natural habitat + (1|SS)+(1|SSB)
	Soy	Log(total abundance) ~ LandUse + CropDiversity + EarthStat yield:% natural habitat + EarthStat yield:GeographicRegion + % natural habitat:LandUse + EarthStat yield + % natural habitat + GeographicRegion + (1|SS) + (1|SSB)
	Wheat	Log(total abundance) ~ GeographicRegion + EarthStat yield + EarthStat yield 10 km buffer-focal difference + % natural habitat + log(annual precipitation) + EarthStat yield:EPIC-log(EPIC BOKU subsistence yield) + EarthStat yield:% natural habitat + EarthStat yield:LandUse + % natural habitat:log(EPIC-BOKU subsistence yield) + log(EPIC-BOKU subsistence yield) + LandUse + (1|SS) + (1|SSB)
	Rice	Log(total abundance) ~ LandUse + log(EarthStat yield) + EPIC-BOKU subsistence yield + log(C - duration human modification) + log(C - annual mean temp resampled) + log(EarthStat yield):% natural habitat + log(EarthStat yield):LandUse + log(EarthStat yield):GeographicRegion + % natural habitat + GeographicRegion + (1|SS) + (1|SSB)
RCAR	Conversion model	RCAR ~ LU-LS categories + GeographicRegion + LU-LS categories: log(C - duration human modification) + LU-LS categories:GeographicRegion + log(C - duration human modification) + (1|SS) + (1|SSB)
	Maize	RCAR ~ LandUse + GeographicRegion + EarthStat yield + % natural habitat+ EarthStat yield:log(EPIC-BOKU subsistence yield) + EarthStat yield:% natural habitat + EarthStat yield:LandUse + EarthStat yield:GeographicRegion + % natural habitat:LandUse + % natural habitat:log(EPIC-BOKU subsistence yield) + log(EPIC-BOKU subsistence yield) + (1|SS) + (1|SSB)
	Soy	RCAR ~ LandUse + GeographicRegion + EPIC-BOKU subsistence yield+ log(C - duration human modification) + EarthStat yield:% natural habitat + EarthStat yield:GeographicRegion + EarthStat yield + % natural habitat (1|SS) + (1|SSB)
	Wheat	RCAR ~ GeographicRegion + % natural habitat + annual mean temperature + % natural habitat:LandUse + % natural habitat: log(EPIC-BOKU subsistence yield) + LandUse + log(EPIC-BOKU subsistence yield) + (1|SS) + (1|SSB)

Response variable	Crop	Formula
	Rice	RCAR ~ LandUse + GeographicRegion + % natural habitat+ log(EarthStat yield): EPIC-BOKU subsistence yield + log(EarthStat yield):% natural habitat + % natural habitat:LandUse + log(EarthStat yield) + EPIC-BOKU subsistence yield + (1|SS) + (1|SSB)

Supplementary Table 8. Summary of the total frequency of selection for continuous variables included in the initial variable set for the yield-biodiversity models for maize, soybean, wheat and rice. For example, percentage of natural habitat was selected 12 times for the 12 models (3 biodiversity metrics for 4 crops) so it was included in all final yield-biodiversity models. Variables were selected through backward stepwise selection based on likelihood-ratio tests with a p-value threshold of 0.05. The default set of initial variables was: EarthStat yield, difference between average yield in 10-km buffer and yield in data location (EarthStat 10-km-buffer yield difference), percentage of natural habitat, difference between average % natural habitat in 10 km buffer and % natural habitat in data location (10-km-buffer % natural habitat difference), EPIC-BOKU subsistence yield, duration of substantial human landscape modification, crop diversity, annual mean temperature, annual precipitation, geographic region (tropical/non-tropical), and local land use as identified in the PREDICTS database.

Variable	Frequency
Percentage of natural habitat	12
Land use	12
EarthStat yield	10
Geographic region (tropical/non-tropical)	10
EPIC-BOKU subsistence yield	8
EarthStat 10-km-buffer yield difference	4
Annual mean temperature	3
Crop diversity	3
Annual precipitation	3
Duration of substantial human landscape modification	3

Model comparisons

Supplementary Table 9 Summary of robustness checks on the yield-biodiversity models in terms of yield and subsistence yield data choices. The column "EarthStat yield + EPIC-Boku subsistence yield" (which are the data sources used for the models presented in the main text) records whether yield, subsistence yield or both were selected in the main models during backward stepwise model selection. The remaining columns record the consistency of modelled results, in terms of magnitude and direction, when different sources of estimates were used for yield and/or subsistence yield. The models are characterised as agreeing on direction if the alternative model results in same-sign biodiversity effects as the reference model. The models are characterised as agreeing on magnitude if the percentage change in a given metric when yield increases by 1 tonne (Supplementary Tables 10-17 and 19) or 0.5 tonne (Supplementary Table 18 due to limited range of data) has the same number of digits before the decimal point as the reference model. The characterisation involves some subjectivity due to interactions in several cases. For detailed information, see the Supplementary Tables 10 – 19

Response variable	Crop	EarthStat yield + EPIC-Boku subsistence yield	MapSPAM yield + EPIC-Boku subsistence yield	EarthStat yield + MapSPAM subsistence yield	MapSPAM yield + MapSPAM subsistence yield
Species richness	Maize	Yield selected	Agree on direction and magnitude	-	-
	Soy	Yield not selected	-	-	-
	Wheat	Yield selected	Agree on direction	-	-
	Rice	Yield and subsistence yield selected	Agree on direction	Agree on direction	Agree on direction
Total abundance	Maize	Yield and subsistence yield selected	Agree on direction	Agree on direction and magnitude	Agree on direction
	Soy	Yield selected	Agree on direction and magnitude	-	-
	Wheat	Yield and subsistence yield selected	Agree on direction	Agree on direction and magnitude	Disagree
	Rice	Yield and subsistence yield selected	Disagree on primary vegetation effect, agree on direction for cropland	Agree on direction and magnitude	Disagree on primary vegetation effect, agree on direction for cropland
RCAR	Maize	Yield and subsistence yield selected	Disagree	Agree on direction and magnitude	Disagree
	Soy	Yield and subsistence yield selected	Agree on direction	Agree on direction and magnitude	Agree on direction
	Wheat	Yield not selected	-	-	-
	Rice	Yield and subsistence yield selected	Agree on direction	Agree on direction and magnitude	Agree on direction

Species Richness

Maize

Supplementary Table 10. Comparison of effect sizes and 95% confidence interval in brackets of the best species richness model for **maize** yield selected with EarthStat yield data and the model fitted with MapSPAM yield data (yield data source indicated in column names, subsistence yield was not selected in the best model). Values represent the percentage difference in species richness when **maize** yields increased from 3 to 4 tonnes/ha and for median values of all other continuous variables in the model. These yield ranges were arbitrarily chosen to sit within the yield levels estimated at the locations of the PREDICTS data and to represent a 1 tonne/ha increase. The existence of only one number across the Primary vegetation/Cropland columns and Tropical/Non-tropical rows indicates that the interactions between yield and land use, and yield and geographic region, respectively, were not selected as significant in the model selection process. The table includes the marginal (R2m) and conditional (R2c) R^2 of the two models. The data sets on which the models were fitted were not identical.

	**Maize**			
	EarthStat		MapSPAM	
	Primary vegetation	Cropland	Primary vegetation	Cropland
Tropical<br>Non-tropical	-7.87% (-8.37%, -7.37%)		-3.69% (-4.13%, -3.25%)	
R2m	0.047		0.036	
R2c	0.98		0.977	

Wheat

Supplementary Table 11. Comparison of effect sizes and 95% confidence interval in brackets of the best species richness model for **wheat** yield selected with EarthStat yield data and EPIC-BOKU subsistence yield, and the models fitted with MapSPAM yield (yield data source in columns, subsistence yield was not selected in the best model). Values represent the percentage difference in species richness when **wheat** yields increased from 3 to 4 tonnes/ha and for median values of all other continuous variables in the model. These yield ranges were arbitrarily chosen to sit within the yield levels estimated at the locations of the PREDICTS data and to represent a 1 tonne/ha increase. The existence of only one number across the Tropical/Non-tropical rows indicates that the interaction between yield and land use was not selected as significant in the model selection process. The table includes the marginal (R2m) and conditional (R2c) R^2 of the models. The data sets on which the models were fitted were not identical.

		**Wheat**			
		EarthStat		MapSPAM	
		Primary vegetation	Cropland	Primary vegetation	Cropland
Boku	Tropical<br>Non-tropical	-16.4% (-17.25%, -15.53%)	0.96% (0.24%, 1.69%)	-1.22% (-1.69%, -0.75%)	-0.23% (-0.48%, 0.02%)
	R2m	0.079		0.087	
	R2c	0.979		0.982	

Rice

Supplementary Table 12. Comparison of effect sizes and 95% confidence interval in brackets of the best species richness model for **rice** yield selected with EarthStat yield data and EPIC-BOKU subsistence yield, and the models fitted with MapSPAM yield and MapSPAM subsistence yield data (yield data source in columns, subsistence yield data source in rows). Values represent the percentage difference in species richness when **rice** yields increased from 3 to 4 tonnes/ha and for median values of all other continuous variables in the model. These yield ranges were arbitrarily chosen to sit within the yield levels estimated at the locations of the PREDICTS data and to represent a 1 tonne/ha increase. The existence of only one number across the Primary vegetation/Cropland columns and Tropical/Non-tropical rows indicates that the interactions between yield and land use, and yield and geographic region, respectively, were not selected as significant in the model selection process. The table includes the marginal (R2m) and conditional (R2c) R^2 of the models. The data sets on which the models were fitted were not identical.

		**Rice**			
		EarthStat		MapSPAM	
		Primary vegetation	Cropland	Primary vegetation	Cropland
Boku	Tropical / Non-tropical	-0.32% (-1.38%, 0.75%)		-4.36% (-4.82%, -3.9%)	
	R2m	0.048		0.042	
	R2c	0.98		0.976	
MapSPAM	Tropical / Non-tropical	-4.66% (-6.13%, -3.16%)		-4.53% (-4.96%, -4.09%)	
	R2m	0.063		0.026	
	R2c	0.977		0.976	

Abundance

Maize

Supplementary Table 13. Comparison of effect sizes and 95% confidence interval in brackets of the best total abundance model for **maize** yield selected with EarthStat yield data and EPIC-BOKU subsistence yield, and the models fitted with MapSPAM yield and MapSPAM subsistence yield data (yield data source in columns, subsistence yield data source in rows). Values represent the percentage difference in total abundance when **maize** yields increased from 3 to 4 tonnes/ha and for median values of all other continuous variables in the model. These yield ranges were arbitrarily chosen to sit within the yield levels estimated at the locations of the PREDICTS data and to represent a 1 tonne/ha increase. The table includes the marginal (R2m) and conditional (R2c) R^2 of the models. The data sets on which the models were fitted were not identical.

		**Maize**			
		EarthStat		MapSPAM	
		Primary vegetation	Cropland	Primary vegetation	Cropland
Boku	Tropical	39.12% (21.34%, 59.5%)	40.83% (24.55%, 59.23%)	4.59% (2.56%, 6.67%)	3.75% (1.81%, 5.72%)
	Non-tropical	-15.99% (-14.73%, -17.24%)	-14.96% (-12.43%, -17.42%)	-2.41% (-1.54%, -3.27%)	-3.2% (-1.03%, -5.32%)
	R2m	0.22		0.109	
	R2c	0.628		0.521	
MapSPAM	Tropical	26.01% (10.52%, 43.68%)	29.72% (15.11%, 46.18%)	4.08% (1.89%, 6.33%)	3.6% (1.53%, 5.71%)
	Non-tropical	-19.1% (-17.6%, -20.57%)	-16.72% (-14.2%, -19.15%)	-3.02% (-1.65%, -4.37%)	-3.47% (-0.98%, -5.9%)
	R2m	0.227		0.11	
	R2c	0.642		0.522	

Soy

Supplementary Table 14. Comparison of effect sizes and 95% confidence interval in brackets of the best total abundance model for **soy** yield selected with EarthStat yield data and EPIC-BOKU subsistence yield, and the models fitted with MapSPAM yield and MapSPAM subsistence yield data (yield data source in columns, subsistence yield data source in rows). Values represent the percentage difference in total abundance when **soy** yields increased from 1.5 to 2.5 tonnes/ha and for median values of all other continuous variables in the model. These yield ranges were arbitrarily chosen to sit within the yield levels estimated at the locations of the PREDICTS data and to represent a 1 tonne/ha increase. The existence of only one number across the Primary vegetation/Cropland columns indicates that the interaction between yield and land use type was not selected as significant in the model selection process. The table includes the marginal (R2m) and conditional (R2c) R^2 of the models. The data sets on which the models were fitted were not identical.

		Soy			
		EarthStat		MapSPAM	
		Primary vegetation	Cropland	Primary vegetation	Cropland
Boku	Tropical	38.72% (38.02%, 39.43%)		76.72% (77.43%, 76.01%)	
	Non-tropical	-36.22% (-34.48%, -37.92%)		-23.08% (-20.04%, -26%)	
	R2m	0.24		0.337	
	R2c	0.564		0.527	

Wheat

Supplementary Table 15. Comparison of effect sizes and 95% confidence interval in brackets of the best total abundance model for **wheat** yield selected with EarthStat yield data and EPIC-BOKU subsistence yield, and the models fitted with MapSPAM yield and MapSPAM subsistence yield data (yield data source in columns, subsistence yield data source in rows). Values represent the percentage difference in total abundance when **wheat** yields increased from 3 to 4 tonnes/ha and for median values of all other continuous variables in the model. These yield ranges were arbitrarily chosen to sit within the yield levels estimated at the locations of the PREDICTS data and to represent a 1 tonne/ha increase. The existence of only one number across the Tropical/Non-tropical rows indicates that the interaction between yield and geographic region was not selected as significant in the model selection process. The table includes the marginal (R2m) and conditional (R2c) R^2 of the models. The data sets on which the models were fitted were not identical. The data sets on which the models were fitted were not identical.

		**Wheat**			
		EarthStat		MapSPAM	
		Primary vegetation	Cropland	Primary vegetation	Cropland
Boku	Tropical<br>Non-tropical	-27.69% (-29.76%, -25.55%)	-6.41% (-7.01%, -5.81%)	-5.86% (-7.38%, -4.32%)	-3.72% (-4.75%, -2.68%)
	R2m	0.122		0.113	
	R2c	0.497		0.49	
MapSPAM	Tropical<br>Non-tropical	-20.56% (-22.27%, -18.82%)	-1.99% (-2.47%, -1.51%)	0.02% (-0.94%, 0.98%)	1.64% (0.81%, 2.47%)
	R2m	0.129		0.187	
	R2c	0.503		0.544	

Rice

Supplementary Table 16. Comparison of effect sizes and 95% confidence interval in brackets of the best total abundance model for **rice** yield selected with EarthStat yield data and EPIC-BOKU subsistence yield, and the models fitted with MapSPAM yield and MapSPAM subsistence yield data (yield data source in columns, subsistence yield data source in rows). Values represent the percentage difference in total abundance when **rice** yields increased from 3 to 4 tonnes/ha and for median values of all other continuous variables in the model. These yield ranges were arbitrarily chosen to sit within the yield levels estimated at the locations of the PREDICTS data and to represent a 1 tonne/ha increase. The existence of only one number across the Tropical/Non-tropical rows indicates that the interaction between yield and geographic region was not selected as significant in the model selection process. The table includes the marginal (R2m) and conditional (R2c) R^2 of the models. The data sets on which the models were fitted were not identical.

		**Rice**			
		EarthStat		MapSPAM	
		Primary vegetation	Cropland	Primary vegetation	Cropland
Boku	Tropical / Non-tropical	8.2% (4.87%, 11.63%)	11.74% (8.89%, 14.67%)	-7.53% (-9.54%, -5.48%)	6.39% (4.5%, 8.32%)
	R2m	0.233		0.204	
	R2c	0.673		0.676	
MapSPAM	Tropical / Non-tropical	5.2% (1.85%, 8.66%)	5.96% (3.64%, 8.34%)	-7.74% (-9.81%, -5.61%)	6.76% (5.33%, 8.21%)
	R2m	0.241		0.204	
	R2c	0.661		0.661	

Relative abundance-weighted average range-size (RCAR)

Maize

Supplementary Table 17. Comparison of effect sizes and 95% confidence interval in brackets of the best RCAR model for **maize** yield selected with EarthStat yield data and EPIC-BOKU subsistence yield, and the models fitted with MapSPAM yield and MapSPAM subsistence yield data (yield data source in columns, subsistence yield data source in rows). Values represent the percentage difference in RCAR when **maize** yields increased from 3 to 4 tonnes/ha and for median values of all other continuous variables in the model. These yield ranges were arbitrarily chosen to sit within the yield levels estimated at the locations of the PREDICTS data and to represent a 1 tonne/ha increase. The table includes the marginal (R2m) and conditional (R2c) R^2 of the models. The data sets on which the models were fitted were not identical.

		**Maize**			
		EarthStat		MapSPAM	
		Primary vegetation	Cropland	Primary vegetation	Cropland
Boku	Tropical	33.09% (12.11%, 58%)	22.28% (3.67%, 44.23%)	-2.76% (-3.89%, -1.62%)	-11.48% (-12.17%, -10.78%)
	Non-tropical	-3.26% (-1.92%, -4.58%)	-11.12% (-9.37%, -12.83%)	1.69% (2.57%, 0.83%)	-7.42% (-6.1%, -8.72%)
	R2m	0.286		0.3	
	R2c	0.956		0.959	
MapSPAM	Tropical	42.63% (21.68%, 67.19%)	29.01% (10.74%, 50.28%)	2.19% (0.58%, 3.83%)	-6.02% (-7.25%, -4.78%)
	Non-tropical	-3.08% (-1.23%, -4.9%)	-12.34% (-10.66%, -13.99%)	5.76% (6.91%, 4.63%)	-2.74% (-1.37%, -4.08%)
	R2m	0.261		0.271	
	R2c	0.957		0.955	

Soy

Supplementary Table 18. Comparison of effect sizes and 95% confidence interval in brackets of the best RCAR model for **soy** yield selected with EarthStat yield data and EPIC-BOKU subsistence yield, and the models fitted with MapSPAM yield and MapSPAM subsistence yield data (yield data source in columns, subsistence yield data source in rows). Values represent the percentage difference in RCAR when **soy** yields increased from 1.5 to 2.5 tonnes/ha and for median values of all other continuous variables in the model. These yield ranges were arbitrarily chosen to sit within the yield levels estimated at the locations of the PREDICTS data and to represent a 1 tonne/ha increase. The table includes the marginal (R2m) and conditional (R2c) R^2 of the models. The data sets on which the models were fitted were not identical.

		**Soy**			
		EarthStat		MapSPAM	
		Primary vegetation	Cropland	Primary vegetation	Cropland
Boku	Tropical	288.91% (268.24%, 310.49%)		56.3% (54.88%, 57.73%)	
	Non-tropical	-32.39% (-31.64%, -33.14%)		-7.2% (-7.24%, -7.17%)	
	R2m	0.352		0.225	
	R2c	0.949		0.926	
MapSPAM	Tropical	806.91% (939.66%, 689.62%)		57.01% (55.33%, 58.7%)	
	Non-tropical	-40.56% (-39.06%, -42.05%)		-6.77% (-5.32%, -8.21%)	
	R2m	0.378		0.224	
	R2c	0.959		0.925	

Rice

Supplementary Table 19. Comparison of effect sizes and 95% confidence interval in brackets of the best RCAR model for **rice** yield selected with EarthStat yield data and EPIC-BOKU subsistence yield, and the models fitted with MapSPAM yield (yield data source in columns, subsistence yield was not selected in the best model). Values represent the percentage difference in RCAR when **rice** yields increased from 3 to 4 tonnes/ha and for median values of all other continuous variables in the model. These yield ranges were arbitrarily chosen to sit within the yield levels estimated at the locations of the PREDICTS data and to represent a 1 tonne/ha increase. The existence of only one number across the Tropical/Non-tropical rows indicates that the interaction between yield and geographic region was not selected as significant in the model selection process. The table includes the marginal (R2m) and conditional (R2c) R^2 of the models. The data sets on which the models were fitted were not identical.

		**Rice**			
		EarthStat		MapSPAM	
		Primary vegetation	Cropland	Primary vegetation	Cropland
Boku	Tropical	10.32% (7.22%, 13.51%)		5.24% (4.24%, 6.25%)	
	Non-tropical				
	R2m	0.24		0.209	
	R2c	0.912		0.92	
MapSPAM	Tropical	10.36% (5.53%, 15.41%)		4.55% (3.35%, 5.75%)	
	Non-tropical				
	R2m	0.287		0.24	
	R2c	0.921		0.921	

Poisson and negative binomial estimate comparison

Supplementary Table 20. Comparison of the results of the species richness models based on Poisson and negative binomial distributions.

Model	Fixed effects	Poisson estimate	Poisson std. error	Neg. binomial estimate	Neg. binomial est. error
Land conversion - Species richness	(Intercept)	2.85	0.10	2.87	0.10
	Primary vegetation - Modified landscape	-0.12	0.04	-0.11	0.03
	Cropland - Natural landscape	-0.28	0.05	-0.27	0.04
	Cropland - Modified landscape	-0.50	0.05	-0.48	0.05
	Geographic region - Tropical	-0.57	0.16	-0.50	0.16
	Annual mean temperature	0.29	0.06	0.24	0.06
	log(annual precipitation)	0.16	0.03	0.17	0.03
	log(C - duration human modification)	0.04	0.02	0.03	0.02
	Primary vegetation - Modified landscape : log(C - duration human modification)	0.00	0.03	0.00	0.03
	Cropland - Natural landscape : log(C - duration human modification)	-0.04	0.05	0.00	0.04
	Cropland - Modified landscape : log(C - duration human modification)	-0.11	0.03	-0.10	0.02
Maize - Species richness	(Intercept)	2.64	0.12	2.66	0.12
	Land Use - Primary vegetation	0.10	0.03	0.16	0.02
	Geographic region - Tropical	-0.33	0.17	-0.42	0.17
	EarthStat yield	-0.26	0.04	-0.29	0.04
	EarthStat yield 10 km buffer-focal difference	-0.04	0.02	-0.03	0.01
	Crop Diversity	-0.08	0.02	-0.06	0.02
	% natural habitat	0.23	0.04	0.35	0.04
	Land Use - Primary vegetation:% natural habitat	-0.12	0.03	-0.25	0.03
	Geographic region - Tropical:% natural habitat	-0.15	0.05	-0.11	0.04
Soy - Species richness	(Intercept)	2.55	0.13	2.49	0.13
	% natural habitat	0.27	0.07	0.22	0.04

Model	Fixed effects	Poisson estimate	Poisson std. error	Neg. binomial estimate	Neg. binomial est. error
	log(Annual precipitation)	0.43	0.11	0.68	0.10
	Land Use - Primary vegetation	0.09	0.05	0.01	0.03
	% natural habitat:Land Use - Primary vegetation	-0.25	0.07	-0.21	0.04
Wheat - Species richness	(Intercept)	2.45	0.11	2.53	0.11
	Geographic region - Tropical	-0.45	0.37	-0.37	0.40
	EarthStat yield	-0.12	0.09	-0.14	0.09
	EarthStat yield 10 km buffer-focal difference	0.04	0.02	0.08	0.02
	% natural habitat	0.23	0.05	0.17	0.05
	Annual mean temperature	0.13	0.06	0.01	0.06
	log(Annual precipitation)	0.22	0.03	0.22	0.03
	Land Use - Primary vegetation	-0.01	0.08	-0.05	0.07
	EarthStat yield : % natural habitat	-0.23	0.05	-0.16	0.04
	EarthStat yield : Land Use - Primary vegetation	-0.17	0.09	-0.19	0.08
	:% natural habitat : Land Use - Primary vegetation	-0.12	0.06	-0.03	0.05
	Geographic region - Tropical:% natural habitat	-0.52	0.20	-0.41	0.23
Rice - Species richness	(Intercept)	2.41	0.10	2.53	0.09
	LandUsePrimary vegetation	0.34	0.04	0.23	0.03
	EPIC-BOKU subsistence yield	-0.09	0.04	-0.09	0.03
	log(EarthStat yield)	0.04	0.05	-0.04	0.05
	% natural habitat	-0.15	0.05	-0.21	0.04
	log(EarthStat yield) : % natural habitat	0.19	0.04	0.12	0.03
	landUsePrimary vegetation : % natural habitat	0.21	0.04	0.25	0.04

Frequentist and Bayesian estimate comparison

Supplementary Table 21. Comparison of the results of yield-biodiversity models run within a Frequentist and a Bayesian framework.

Model	Fixed effects	Frequentist estimate	Frequentist std. error	Bayesian estimate	Bayesian est. error
Land conversion - Species richness	Intercept	2.85	0.10	2.85	0.10
	Primary vegetation - Modified landscape	-0.12	0.04	-0.12	0.04
	Cropland - Natural landscape	-0.28	0.05	-0.28	0.05
	Cropland - Modified landscape	-0.50	0.05	-0.50	0.05
	Geographic region - Tropical	-0.57	0.16	-0.58	0.16
	Annual mean temperature	0.29	0.06	0.30	0.06
	log(annual precipitation)	0.16	0.03	0.16	0.03
	log(C - duration human modification)	0.04	0.02	0.04	0.02
	Primary vegetation - Modified landscape : log(C - duration human modification)	0.00	0.03	0.00	0.03
	Cropland - Natural landscape : log(C - duration human modification)	-0.04	0.05	-0.04	0.05
	Cropland - Modified landscape : log(C - duration human modification)	-0.11	0.03	-0.11	0.03
Maize - Species richness	Intercept	2.64	0.12	2.63	0.12
	Land Use - Primary vegetation	0.10	0.03	0.10	0.03
	Geographic region - Tropical	-0.33	0.17	-0.35	0.17
	EarthStat yield	-0.26	0.04	-0.26	0.04
	EarthStat yield 10 km buffer-focal difference	-0.04	0.02	-0.04	0.02
	Crop Diversity	-0.08	0.02	-0.08	0.02
	% natural habitat	0.23	0.04	0.23	0.05
	Land Use - Primary vegetation : % natural habitat	-0.12	0.03	-0.11	0.04
	Geographic region – Tropical : % natural habitat	-0.15	0.05	-0.15	0.05
Soy - Species richness	Intercept	2.55	0.13	2.54	0.13
	% natural habitat	0.27	0.07	0.27	0.07
	log(annual precipitation)	0.43	0.11	0.43	0.11
	Land Use - Primary vegetation	0.09	0.05	0.09	0.05

Model	Fixed effects	Frequentist estimate	Frequentist std. error	Bayesian estimate	Bayesian est. error
	% natural habitat:Land Use - Primary vegetation	-0.25	0.07	-0.25	0.07
Wheat - Species richness	Intercept	2.45	0.11	2.45	0.12
	Geographic region - Tropical	-0.45	0.37	-0.46	0.38
	EarthStat yield	-0.12	0.09	-0.12	0.09
	EarthStat yield 10 km buffer-focal difference	0.04	0.02	0.04	0.02
	% natural habitat	0.23	0.05	0.23	0.05
	Annual mean temperature	0.13	0.06	0.13	0.06
	log(annual precipitation)	0.22	0.03	0.22	0.03
	Land Use - Primary vegetation	-0.01	0.08	-0.01	0.08
	EarthStat yield : % natural habitat	-0.23	0.05	-0.24	0.05
	EarthStat yield : Land Use - Primary vegetation	-0.17	0.09	-0.17	0.09
	% natural habitat : Land Use - Primary vegetation	-0.12	0.06	-0.12	0.06
	Geographic region - Tropical:% natural habitat	-0.52	0.20	-0.52	0.20
Rice - Species richness	Intercept	2.41	0.10	2.41	0.10
	Land Use - Primary vegetation	0.34	0.04	0.34	0.04
	EPIC-BOKU subsistence yield	-0.09	0.04	-0.09	0.04
	log(EarthStat yield)	0.04	0.05	0.04	0.05
	% natural habitat	-0.15	0.05	-0.15	0.05
	log(EarthStat yield) : % natural habitat	0.19	0.04	0.19	0.04
	Land Use - Primary vegetation : % natural habitat	0.21	0.04	0.21	0.04
Land conversion - Total abundance	Intercept	-0.53	0.09	-0.54	0.09
	Primary vegetation - Modified landscape	-0.45	0.10	-0.45	0.10
	Cropland - Natural landscape	-0.27	0.16	-0.27	0.16
	Cropland - Modified landscape	-0.61	0.10	-0.61	0.10
	Geographic region - Tropical	-0.37	0.15	-0.37	0.15
	Annual mean temperature	0.29	0.07	0.29	0.07

Model	Fixed effects	Frequentist estimate	Frequentist std. error	Bayesian estimate	Bayesian est. error
	Primary vegetation - Modified landscape:Geographic region - Tropical	0.37	0.16	0.37	0.16
	Cropland - Natural landscape:Geographic region - Tropical	-0.10	0.19	-0.10	0.19
	Cropland - Modified landscape:Geographic region - Tropical	-0.41	0.21	-0.42	0.21
Maize - Total abundance	Intercept	-0.92	0.09	-0.91	0.10
	Land Use - Primary vegetation	0.22	0.05	0.22	0.05
	Geographic region - Tropical	0.83	0.28	0.86	0.28
	EarthStat yield	-0.41	0.08	-0.41	0.08
	EarthStat yield 10 km buffer-focal difference	-0.16	0.03	-0.16	0.03
	Crop Diversity	-0.12	0.04	-0.13	0.04
	log(C - duration human modification)	0.17	0.05	0.17	0.05
	log(EPIC-BOKU subsistence yield)	0.17	0.07	0.17	0.07
	% natural habitat	0.10	0.05	0.10	0.05
	EarthStat yield : log(EPIC-BOKU subsistence yield)	0.16	0.07	0.16	0.07
	EarthStat yield : % natural habitat	0.16	0.05	0.16	0.05
	Land Use - Primary vegetation : EarthStat yield	-0.24	0.05	-0.24	0.06
	Geographic region - Tropical : EarthStat yield	1.61	0.27	1.65	0.28
Soy - Total abundance	Intercept	-0.98	0.12	-0.98	0.13
	Land Use - Primary vegetation	0.28	0.07	0.28	0.07
	Crop Diversity	0.15	0.05	0.16	0.05
	EarthStat yield	-0.23	0.09	-0.24	0.09
	% natural habitat	0.37	0.08	0.38	0.08
	Geographic region - Tropical	0.02	0.20	0.03	0.20
	EarthStat yield : % natural habitat	0.17	0.07	0.18	0.07
	EarthStat yield : Geographic region - Tropical	0.44	0.17	0.46	0.18

Model	Fixed effects	Frequentist estimate	Frequentist std. error	Bayesian estimate	Bayesian est. error
	Land Use - Primary vegetation : % natural habitat	-0.42	0.09	-0.43	0.09
Wheat - Total abundance	Intercept	-1.05	0.11	-1.04	0.11
	Geographic region - Tropical	-1.57	0.53	-1.58	0.54
	EarthStat yield	-0.27	0.10	-0.27	0.11
	EarthStat yield 10 km buffer-focal difference	0.07	0.03	0.07	0.03
	% natural habitat	-0.01	0.06	-0.01	0.06
	log(annual precipitation)	0.40	0.05	0.40	0.05
	log(EPIC-BOKU subsistence yield)	-0.05	0.06	-0.05	0.07
	Land Use - Primary vegetation	-0.22	0.14	-0.23	0.14
	EarthStat yield : log(EPIC-BOKU subsistence yield)	-0.14	0.06	-0.14	0.06
	EarthStat yield : % natural habitat	-0.23	0.07	-0.23	0.07
	EarthStat yield : Land Use - Primary vegetation	-0.43	0.16	-0.45	0.17
	% natural habitat : log(EPIC-BOKU subsistence yield)	-0.08	0.03	-0.08	0.03
Rice - Total abundance	Intercept	-1.15	0.24	-1.15	0.24
	Land Use - Primary vegetation	0.54	0.06	0.55	0.06
	log(EarthStat yield)	-0.15	0.19	-0.14	0.19
	EPIC-BOKU subsistence yield	-0.23	0.04	-0.23	0.04
	log(C - duration human modification)	0.27	0.06	0.28	0.06
	log(C - annual precipitation)	0.25	0.09	0.25	0.09
	% natural habitat	0.01	0.05	0.01	0.05
	Geographic region - Tropical	-0.03	0.26	-0.02	0.26
	log(EarthStat yield) : % natural habitat	0.11	0.06	0.11	0.06
	Land Use - Primary vegetation : log(EarthStat yield)	-0.23	0.05	-0.22	0.05
	log(EarthStat yield) : Geographic region - Tropical	0.47	0.21	0.47	0.21
Land conversion - RCAR	Intercept	5.53	0.06	5.54	0.06
	Primary vegetation - Modified landscape	0.11	0.06	0.11	0.06

Model	Fixed effects	Frequentist estimate	Frequentist std. error	Bayesian estimate	Bayesian est. error
	Cropland - Natural landscape	0.10	0.06	0.10	0.06
	Cropland - Modified landscape	0.23	0.06	0.23	0.06
	Geographic region - Tropical	-0.60	0.08	-0.60	0.09
	log(C - duration human modification)	0.05	0.03	0.04	0.03
	Primary vegetation - Modified landscape : log(C - duration human modification)	-0.03	0.05	-0.03	0.05
	Cropland - Natural landscape : log(C - duration human modification)	-0.09	0.03	-0.09	0.03
	Cropland - Modified landscape : log(C - duration human modification)	-0.01	0.05	-0.01	0.05
	Primary vegetation - Modified landscape : Geographic region - Tropical	-0.08	0.07	-0.07	0.07
	Cropland - Natural landscape : Geographic region - Tropical	0.31	0.07	0.31	0.07
	Cropland - Modified landscape : Geographic region - Tropical	-0.02	0.12	-0.02	0.12
Maize - RCAR	Intercept	5.87	0.08	5.86	0.08
	Land Use - Primary vegetation	-0.16	0.01	-0.16	0.01
	Geographic region - Tropical	-0.33	0.20	-0.33	0.20
	EarthStat yield	-0.15	0.04	-0.15	0.04
	% natural habitat	-0.07	0.02	-0.07	0.02
	log(EPIC-BOKU subsistence yield)	-0.08	0.04	-0.08	0.04
	EarthStat yield : log(EPIC-BOKU subsistence yield)	-0.06	0.03	-0.07	0.03
	EarthStat yield : % natural habitat	0.06	0.02	0.06	0.02
	Land Use - Primary vegetation : EarthStat yield	0.12	0.01	0.12	0.01
	Geographic region - Tropical : EarthStat yield	0.44	0.16	0.45	0.17
	Land Use - Primary vegetation : % natural habitat	0.04	0.02	0.04	0.02
	% natural habitat : log(EPIC-BOKU subsistence yield)	0.06	0.01	0.07	0.01
Soy - RCAR	Intercept	5.73	0.13	5.74	0.14

Model	Fixed effects	Frequentist estimate	Frequentist std. error	Bayesian estimate	Bayesian est. error
	Land Use - Primary vegetation	-0.14	0.02	-0.13	0.02
	Geographic region - Tropical	-0.49	0.21	-0.48	0.22
	EPIC-BOKU subsistence yield	-0.03	0.01	-0.03	0.01
	log(C - duration human modification)	-0.03	0.06	-0.04	0.06
	EarthStat yield	-0.08	0.02	-0.08	0.03
	% natural habitat	0.00	0.02	-0.01	0.02
	EarthStat yield : % natural habitat	0.09	0.02	0.09	0.03
	Geographic region - Tropical : EarthStat yield	0.55	0.16	0.59	0.17
Wheat - RCAR	Intercept	5.91	0.07	5.91	0.07
	Geographic region - Tropical	-0.77	0.25	-0.78	0.26
	% natural habitat	-0.01	0.02	-0.01	0.02
	Annual mean temperature	0.07	0.05	0.07	0.05
	Land Use - Primary vegetation	-0.05	0.03	-0.05	0.03
	log(EPIC-BOKU subsistence yield)	-0.03	0.03	-0.03	0.03
	% natural habitat : Land Use - Primary vegetation	-0.08	0.03	-0.08	0.03
	% natural habitat : log(EPIC-BOKU subsistence yield)	0.03	0.02	0.03	0.02
Rice - RCAR	Intercept	5.42	0.14	5.42	0.14
	Land Use - Primary vegetation	-0.27	0.03	-0.27	0.03
	Geographic region - Tropical	-0.29	0.16	-0.29	0.16
	% natural habitat	0.03	0.03	0.03	0.03
	log(EarthStat yield)	0.02	0.04	0.02	0.04
	EPIC-BOKU subsistence yield	-0.05	0.03	-0.05	0.03
	log(EarthStat yield) : EPIC-BOKU subsistence yield	-0.07	0.04	-0.07	0.04
	% natural habitat : log(EarthStat yield)	-0.17	0.04	-0.17	0.04
	Land Use - Primary vegetation : % natural habitat	-0.13	0.03	-0.13	0.03

Supplementary figures

Data points distribution

Supplementary Figure 1. Global distribution of PREDICTS studies used in the analysis of the effect of EarthStat **maize** yield on local species richness. Red dots indicate tropical sites and blue dots indicate non-tropical sites.

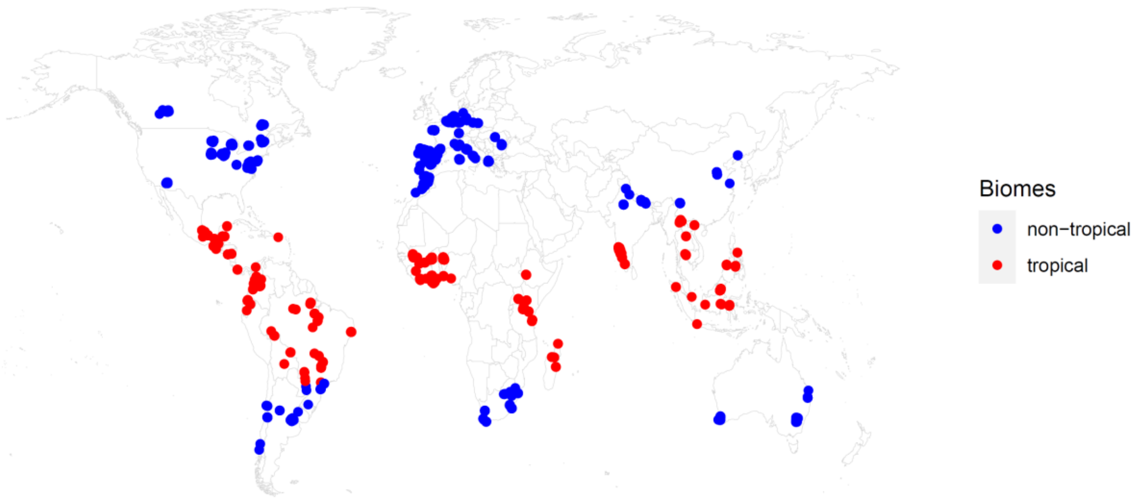

Supplementary Figure 2. Global distribution of PREDICTS studies used in the analysis of the effect of EarthStat **soy** yield on local species richness. Red dots indicate tropical sites and blue dots indicate non-tropical sites.

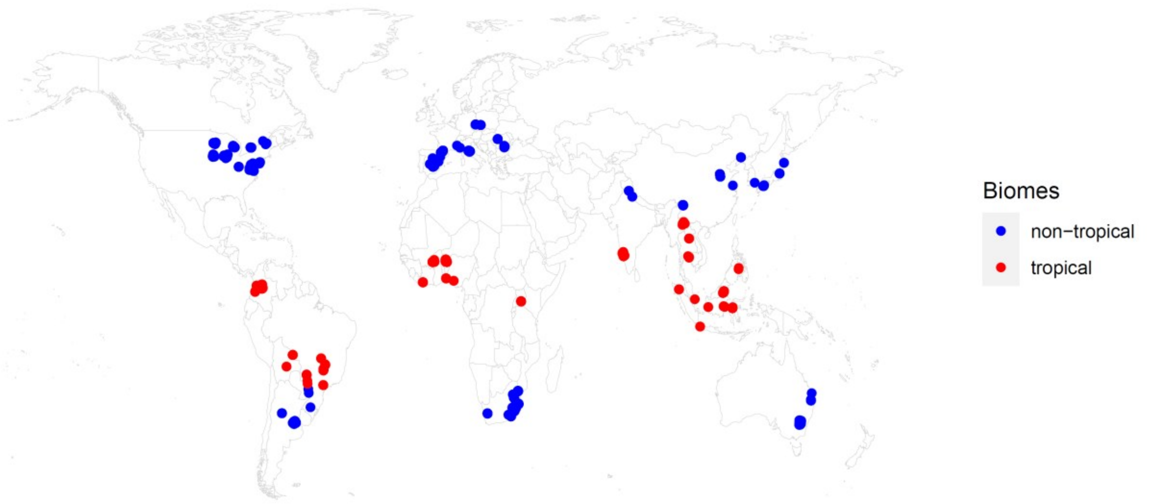

Supplementary Figure 3. Global distribution of PREDICTS studies used in the analysis of the effect of EarthStat **wheat** yield on local species richness. Red dots indicate tropical sites and blue dots indicate non-tropical sites.

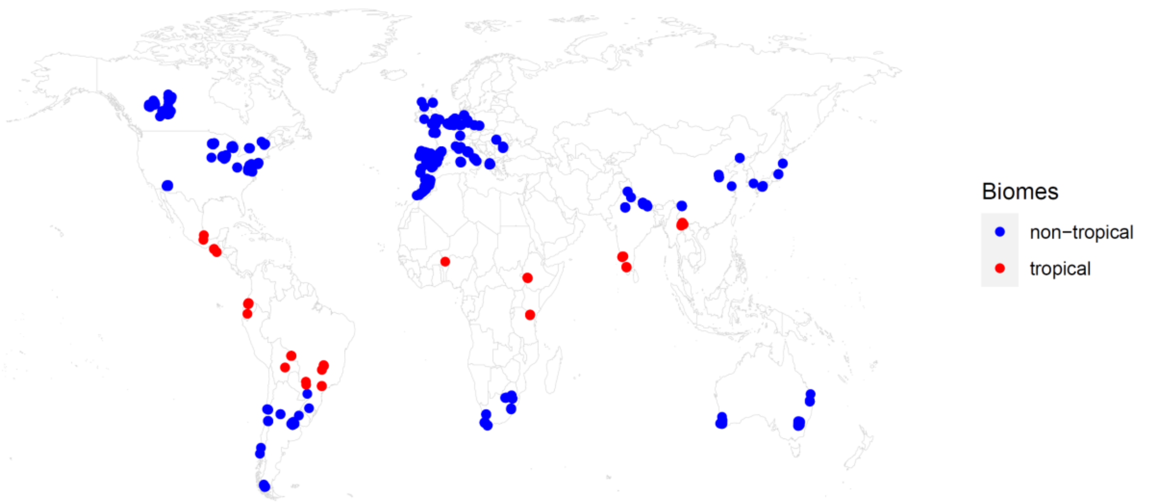

Supplementary Figure 4. Global distribution of PREDICTS studies used in the analysis of the effect of EarthStat **rice** yield on local species richness. Red dots indicate tropical sites and blue dots indicate non-tropical sites.

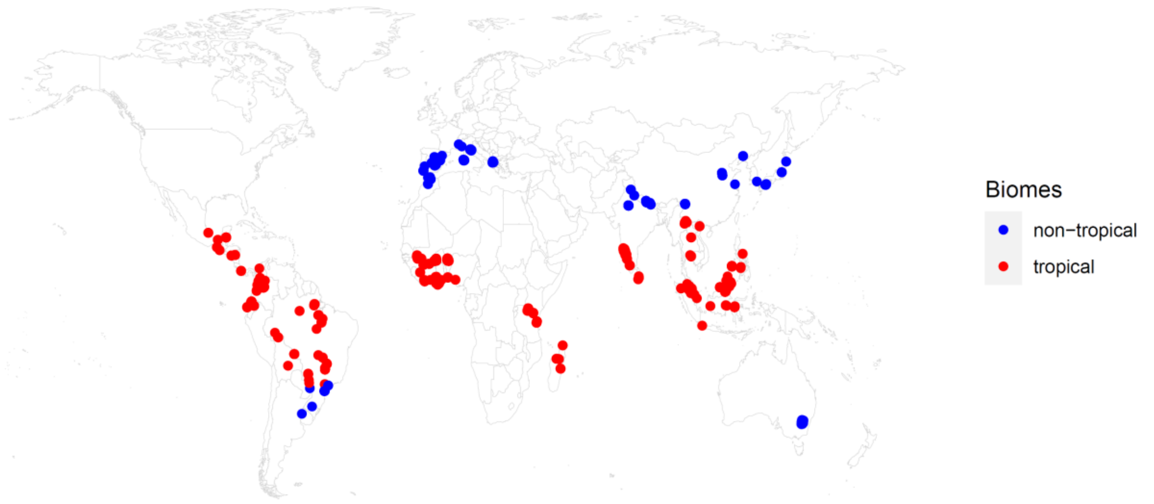

Model checks

Species richness – Poisson models

Supplementary Figure 5. Model checks for the land conversion - species richness model fitted with a Poisson distribution. The Q-Q plot (left panel) and the plot of residuals against the predicted values (right panel) were created with the DHARMa R package, which uses a simulation based approach to standardise residuals of generalised mixed-effects models. In the right panel, the solid red line indicates the expected and the dashed red line represents the observed 0.5 quantiles in the y direction of the distribution of simulated residuals. The weights used in the initial model are ignored.

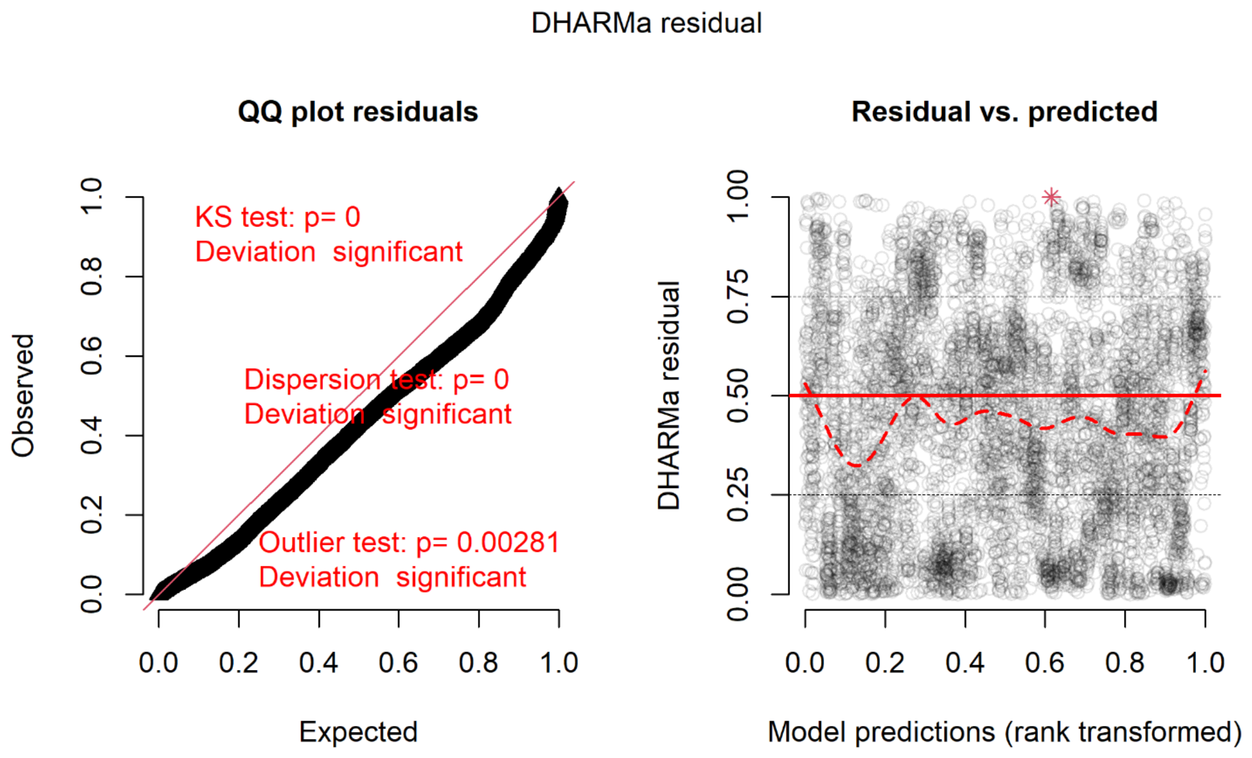

Supplementary Figure 6. Model checks for the maize-species richness model fitted with a Poisson distribution. The Q-Q plot (left panel) and the plot of residuals against the predicted values (right panel) were created with the DHARMa R package, which uses a simulation based approach to standardise residuals of generalised mixed-effects models. In the right panel, the solid red line indicates the expected and the dashed red line represents the observed 0.5 quantiles in the y direction of the distribution of simulated residuals. The weights used in the initial model are ignored.

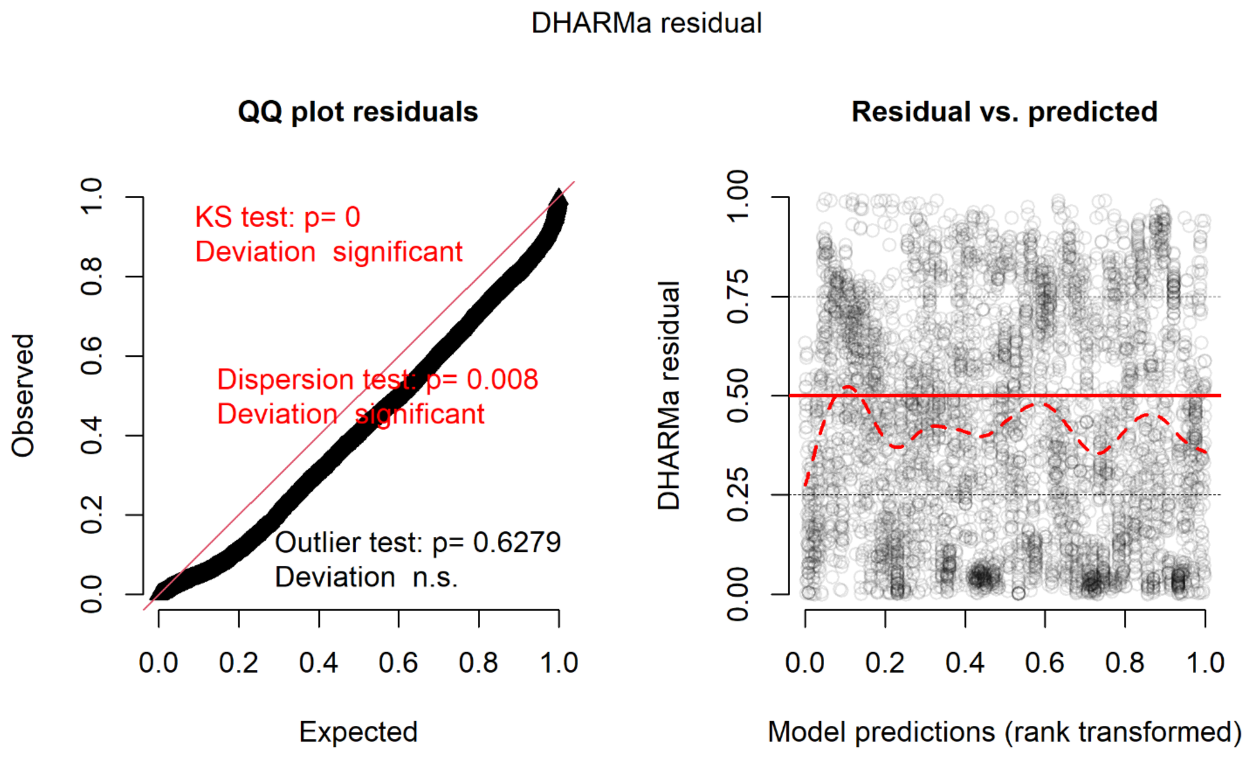

Supplementary Figure 7. Model checks for the soy-species richness model fitted with a Poisson distribution. The Q-Q plot (left panel) and the plot of residuals against the predicted values (right panel) were created with the DHARMa R package, which uses a simulation based approach to standardise residuals of generalised mixed-effects models. In the right panel, the solid red line indicates the expected and the dashed red line represents the observed 0.5 quantiles in the y direction of the distribution of simulated residuals. The weights used in the initial model are ignored.

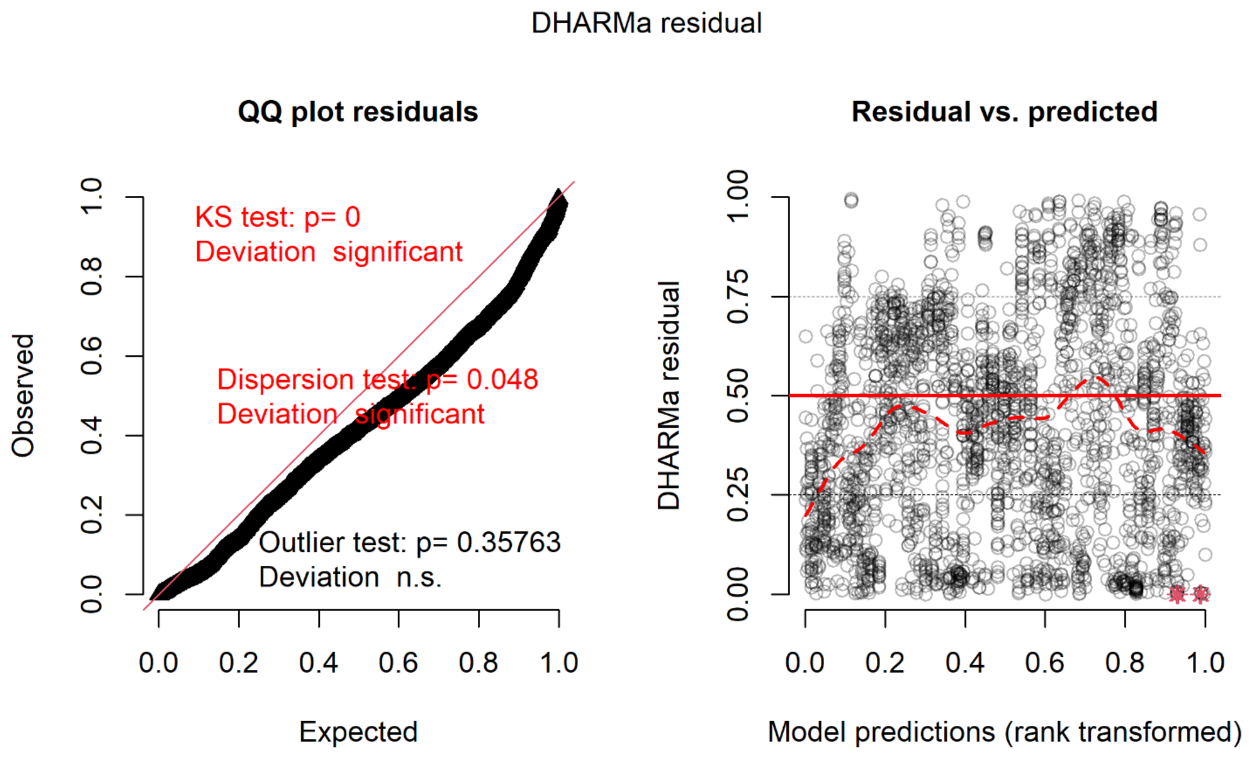

Supplementary Figure 8. Model checks for the wheat-species richness model fitted with a Poisson distribution. The Q-Q plot (left panel) and the plot of residuals against the predicted values (right panel) were created with the DHARMa R package, which uses a simulation based approach to standardise residuals of generalised mixed-effects models. In the right panel, the solid red line indicates the expected and the dashed red line represents the observed 0.5 quantiles in the y direction of the distribution of simulated residuals. The weights used in the initial model are ignored.

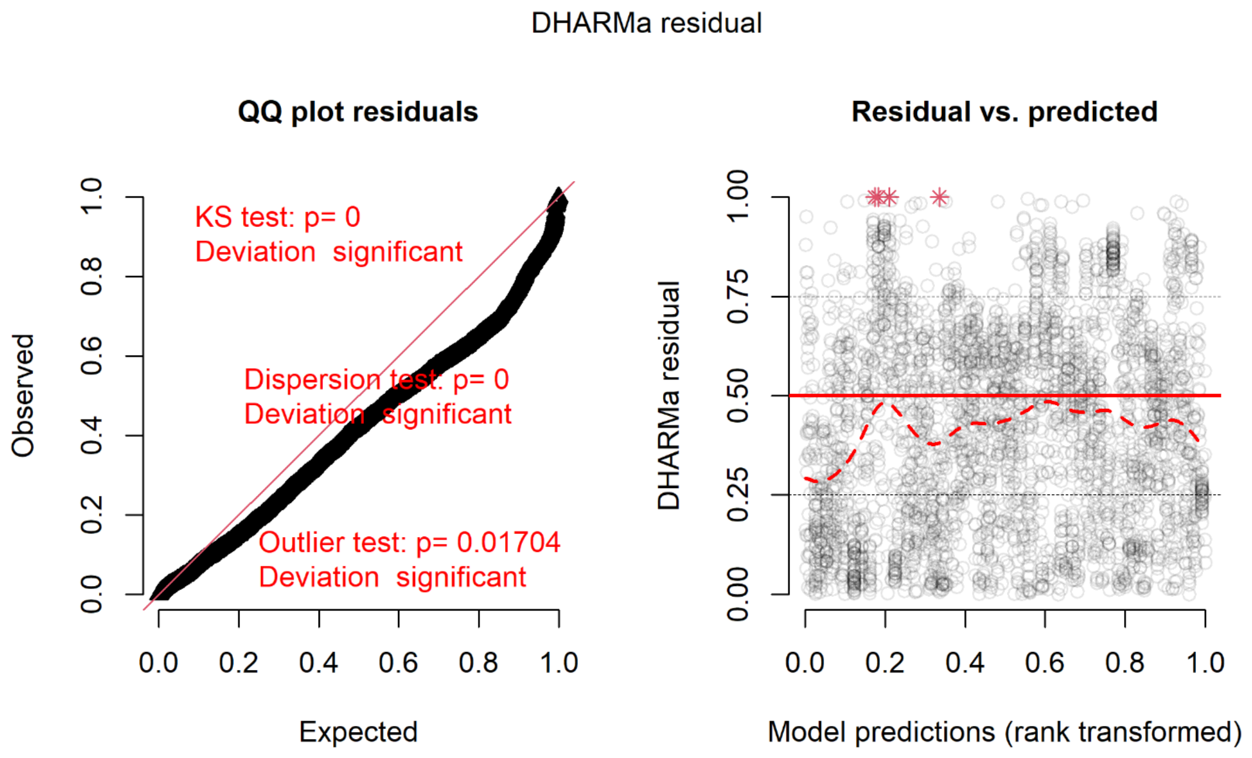

Supplementary Figure 9. Model checks for the rice-species richness model fitted with a Poisson distribution. The Q-Q plot (left panel) and the plot of residuals against the predicted values (right panel) were created with the DHARMa R package, which uses a simulation based approach to standardise residuals of generalised mixed-effects models. In the right panel, the solid red line indicates the expected and the dashed red line represents the observed 0.5 quantiles in the y direction of the distribution of simulated residuals. The weights used in the initial model are ignored.

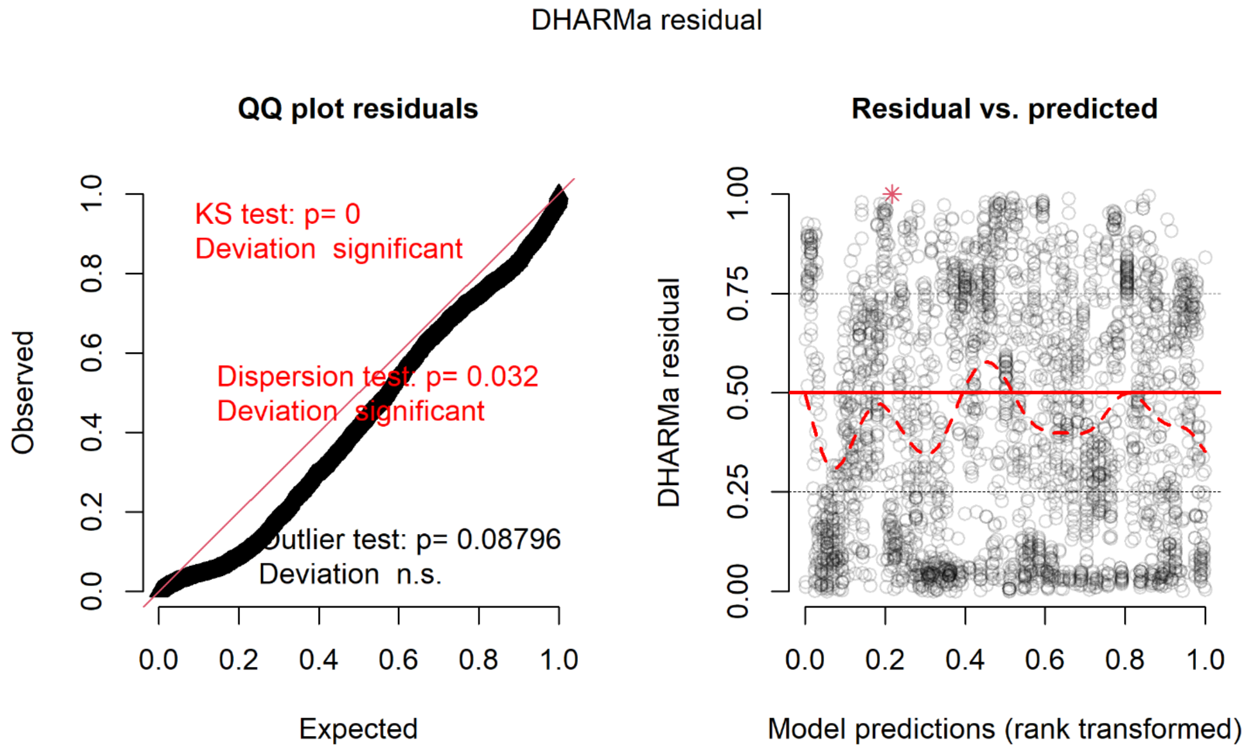

Species richness – Negative binomial models

Supplementary Figure 10. Model checks for the land conversion-species richness model fitted with a negative binomial distribution. The Q-Q plot (left panel) and the plot of residuals against the predicted values (right panel) were created with the DHARMa R package, which uses a simulation based approach to standardise residuals of generalised mixed-effects models. In the right panel, the solid red line indicates the expected and the dashed red line represents the observed 0.5 quantiles in the y direction of the distribution of simulated residuals. The weights used in the initial model are ignored.

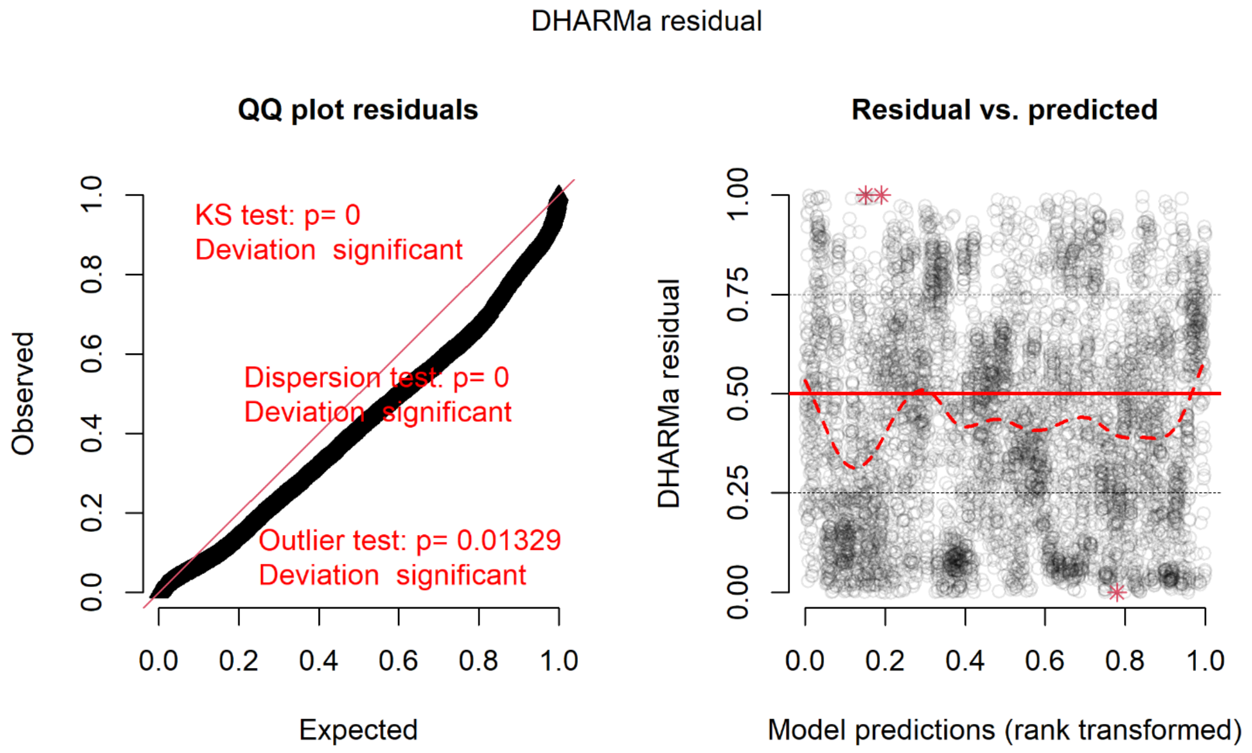

Supplementary Figure 11. Model checks for the maize-species richness model fitted with a negative binomial distribution. The Q-Q plot (left panel) and the plot of residuals against the predicted values (right panel) were created with the DHARMa R package, which uses a simulation based approach to standardise residuals of generalised mixed-effects models. In the right panel, the solid red line indicates the expected and the dashed red line represents the observed 0.5 quantiles in the y direction of the distribution of simulated residuals. The weights used in the initial model are ignored.

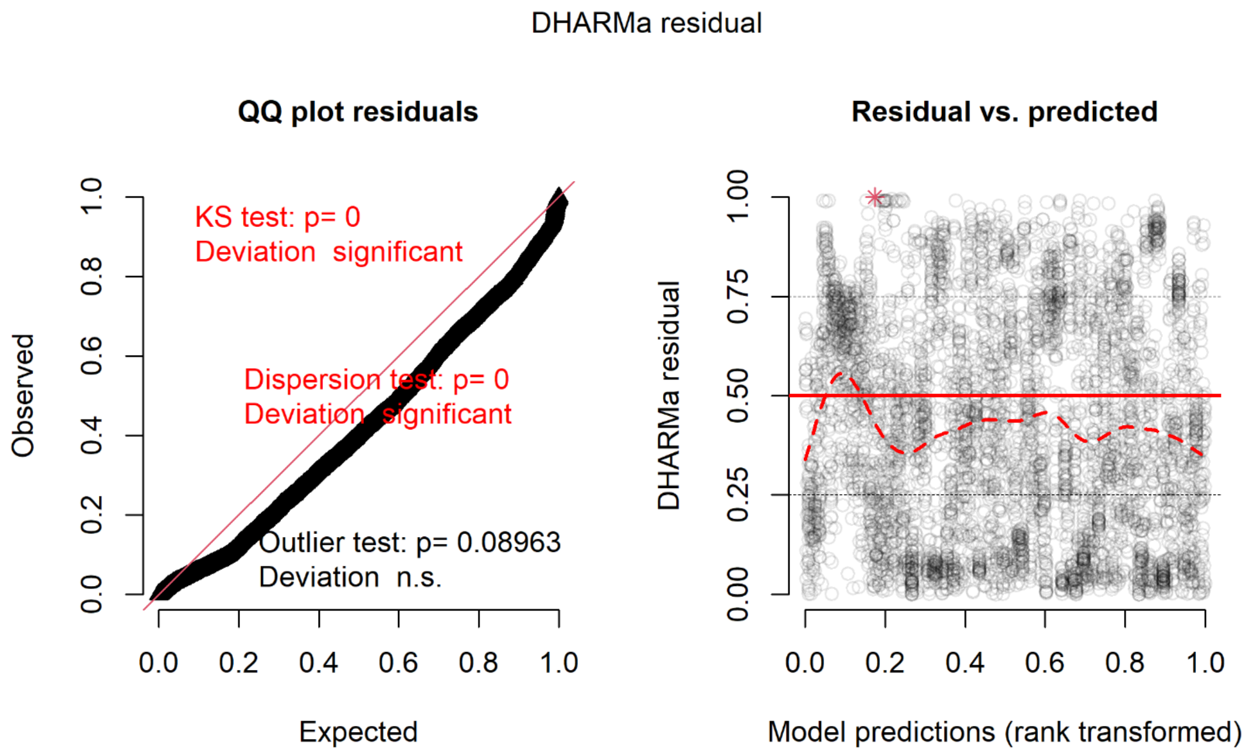

Supplementary Figure 12. Model checks for the soy-species richness model fitted with a negative binomial distribution. The Q-Q plot (left panel) and the plot of residuals against the predicted values (right panel) were created with the DHARMa R package, which uses a simulation based approach to standardise residuals of generalised mixed-effects models. In the right panel, the solid red line indicates the expected and the dashed red line represents the observed 0.5 quantiles in the y direction of the distribution of simulated residuals. The weights used in the initial model are ignored.

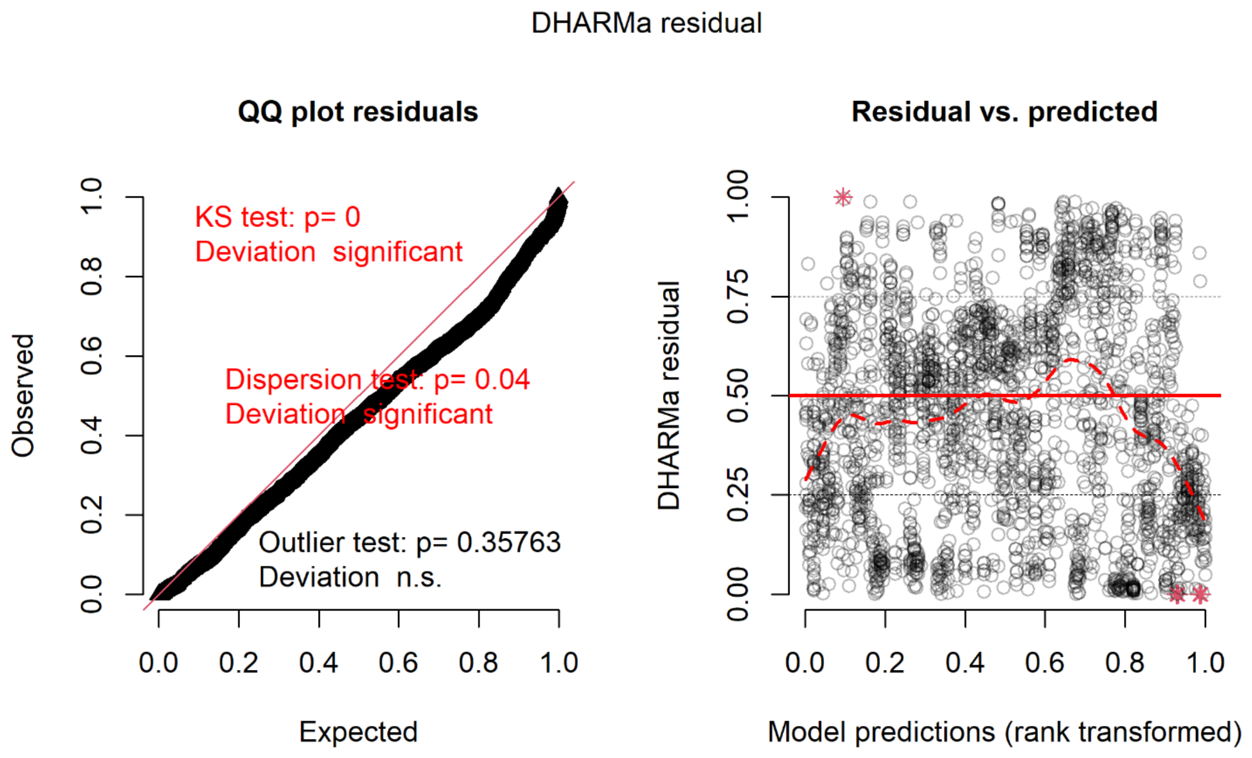

Supplementary Figure 13. Model checks for the wheat-species richness model fitted with a negative binomial distribution. The Q-Q plot (left panel) and the plot of residuals against the predicted values (right panel) were created with the DHARMa R package, which uses a simulation based approach to standardise residuals of generalised mixed-effects models. In the right panel, the solid red line indicates the expected and the dashed red line represents the observed 0.5 quantiles in the y direction of the distribution of simulated residuals. The weights used in the initial model are ignored.

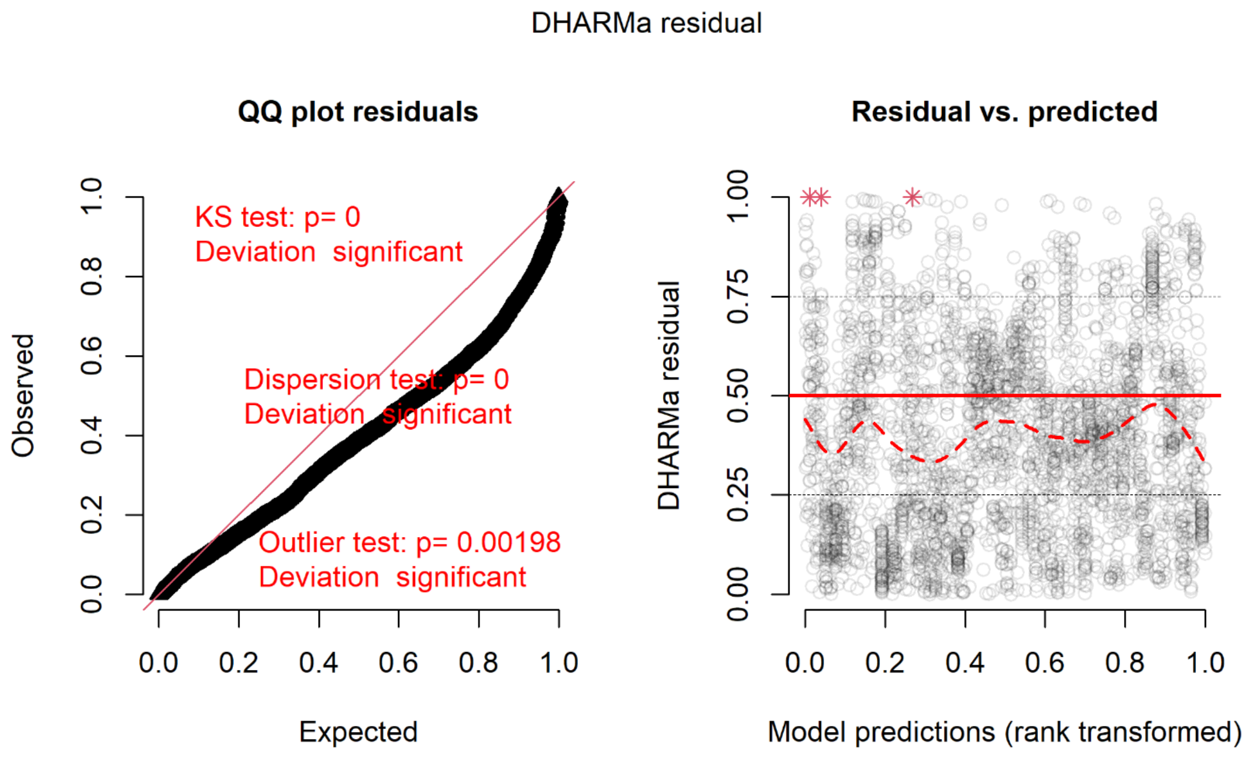

Supplementary Figure 14. Model checks for the rice-species richness model fitted with a negative binomial distribution. The Q-Q plot (left panel) and the plot of residuals against the predicted values (right panel) were created with the DHARMa R package, which uses a simulation based approach to standardise residuals of generalised mixed-effects models. In the right panel, the solid red line indicates the expected and the dashed red line represents the observed 0.5 quantiles in the y direction of the distribution of simulated residuals. The weights used in the initial model are ignored.

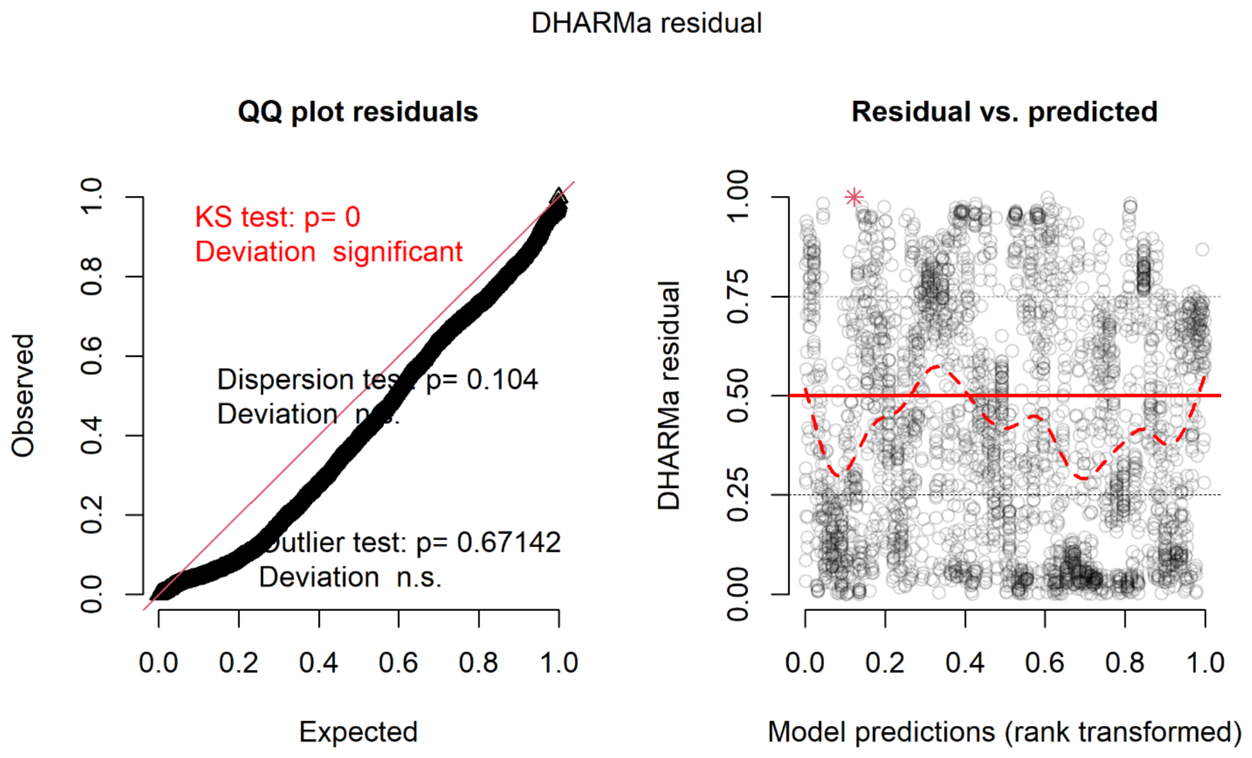

Supplementary Figure 15. The effect of increasing the yield a) maize, b) soy, c) wheat, and d) rice on species richness calculated based on models assuming a negative binomial distribution. The modelled species richness are based on models using EarthStat estimates of actual yields. The number of data points for each model can be found in Supplementary Tables 1-5. The plots show, where selected in the final model, the responses of biodiversity metrics to interactions between yield and the following variables: geographic region–- red hues for tropical regions, and grey hues if geographic region was not significant either on its own or in interactions; percentage of natural habitat–- dark hues for 85% natural habitat and light hues for 15% natural habitat. Where geographic region on its own was selected in the model but the interaction with yield was not selected in the final model, we plotted the values for the geographic region with most data points. Where land use on its own was selected in the model but the interaction with yield was not selected in the final model, we plotted the values for the land use type with most data points. Where percentage of natural vegetation on its own was selected in the model but the interaction with yield was not selected in the final model, we plotted the values for low percentage of natural vegetation (15% natural habitat). For cases where yield was not selected in the best model, the plot was left empty. The lines represent median predicted values and shaded areas represent 95% confidence intervals. The ticks at the bottom of each plot illustrate the yield values in each dataset rescaled to a [0, 1] interval, with red for tropical regions and blue for non-tropical regions. The plots illustrate model predictions that do not necessarily always represent plausible combinations of yield and other variable values. The biodiversity metrics were scaled to the level of the respective metric in primary vegetation in agricultural landscapes with the lowest yield among all the sites in the analysis, a value of 0% meaning that the biodiversity level has not changed compared to biodiversity in primary vegetation at the lowest yield.

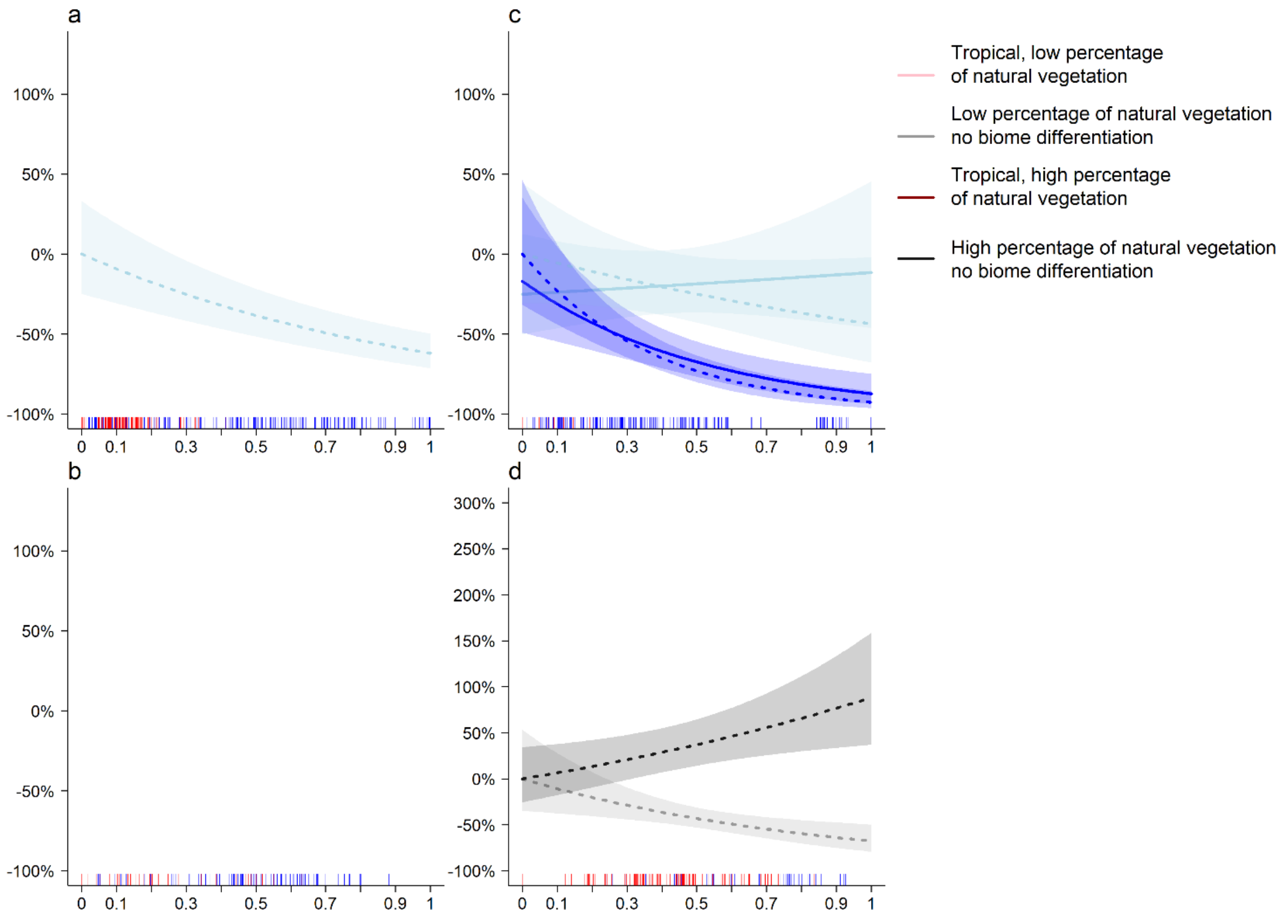

Total abundance

Supplementary Figure 16. Model checks for the land conversion-total abundance model. a. Plot of fitted values vs. Pearson residuals b. Q-Q plot to check the normality of the residuals. c. Plot of observed vs predicted values.

a.

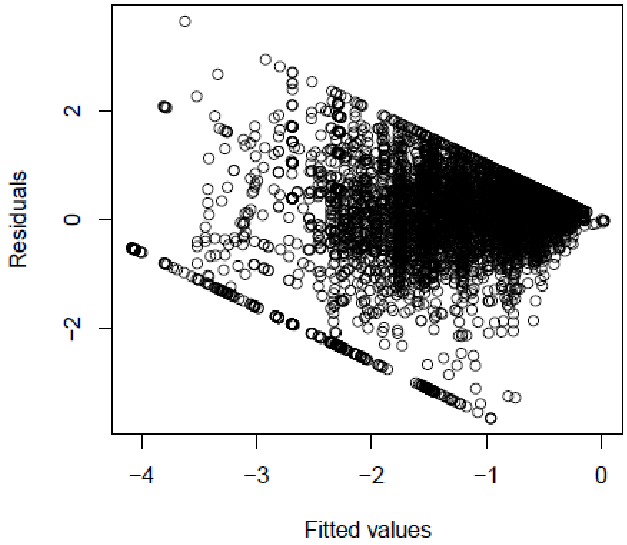

b.

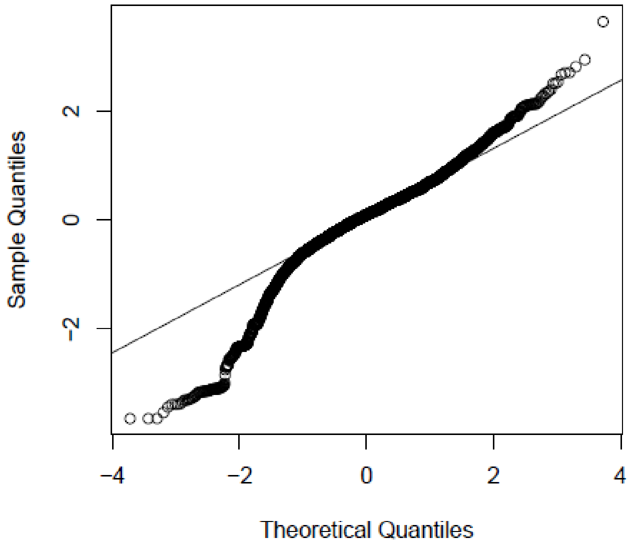

c.

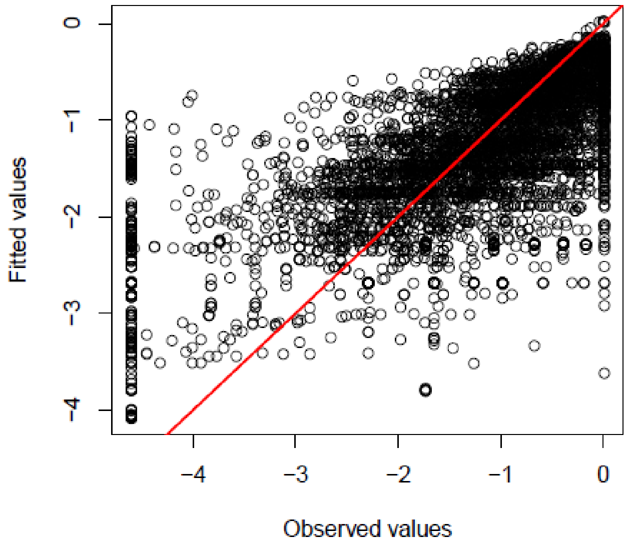

Supplementary Figure 17. Model checks for the maize-total abundance model. a. Plot of fitted values vs. Pearson residuals b. Q-Q plot to check the normality of the residuals. c. Plot of observed vs predicted values.

a

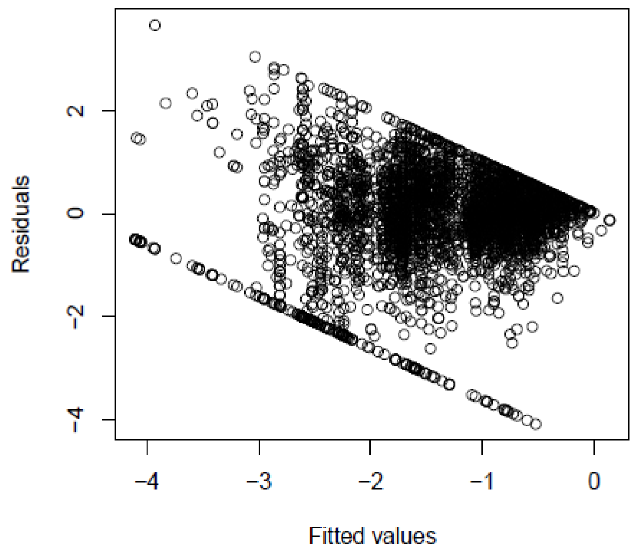

b

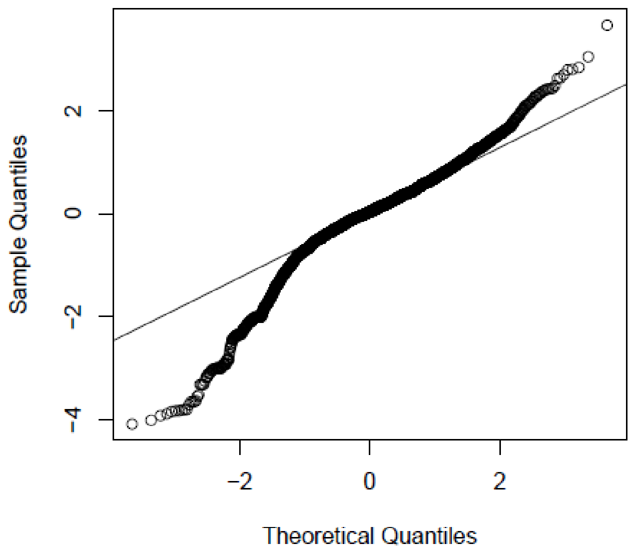

c

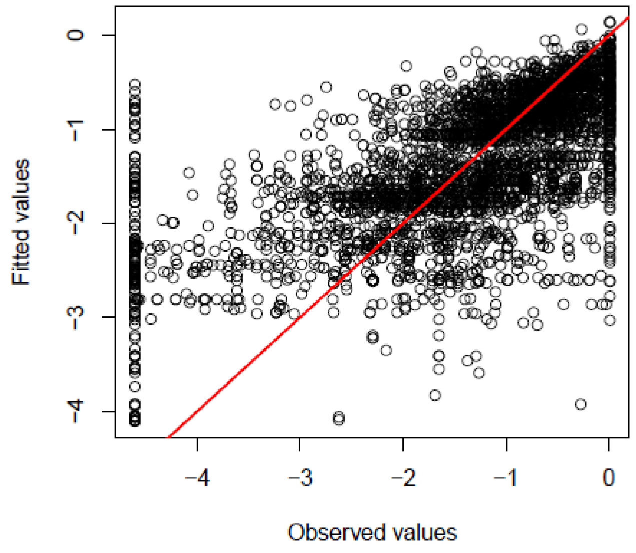

Supplementary Figure 18. Model checks for the soy-total abundance model. a. Plot of fitted values vs. Pearson residuals b. Q-Q plot to check the normality of the residuals. c. Plot of observed vs predicted values.

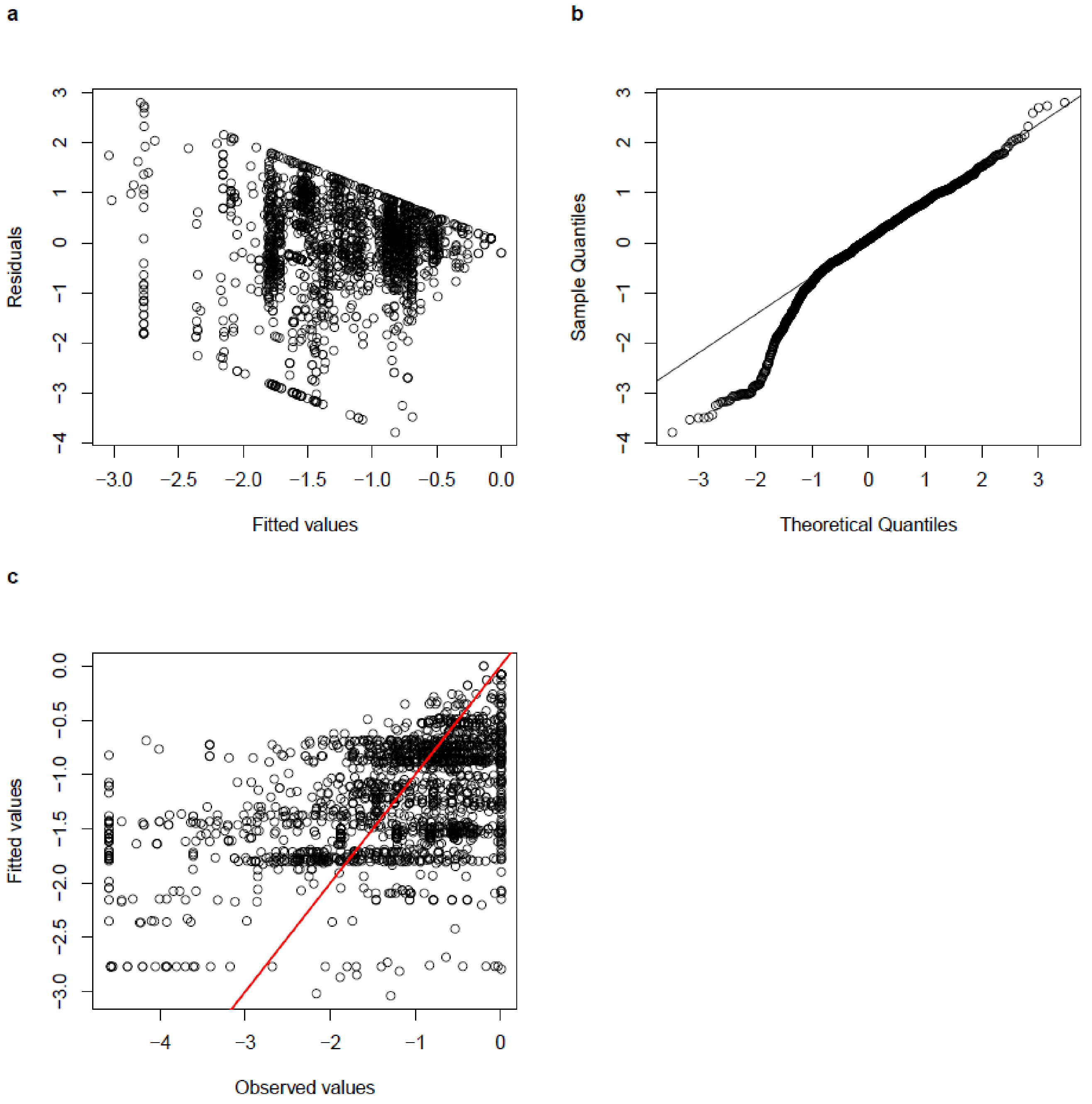

Supplementary Figure 19. Model checks for the wheat-total abundance model. a. Plot of fitted values vs. Pearson residuals b. Q-Q plot to check the normality of the residuals. c. Plot of observed vs predicted values.

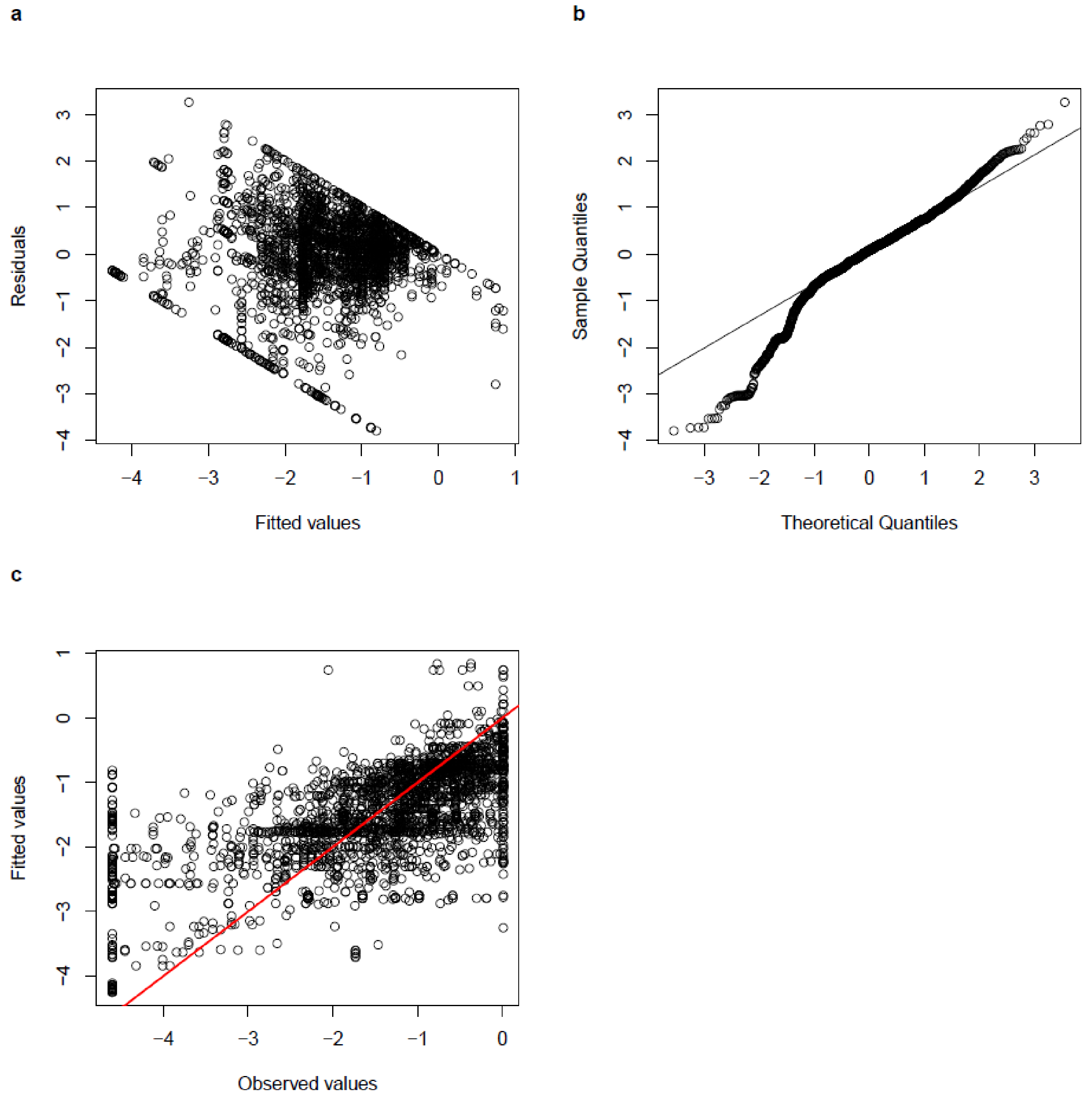

Supplementary Figure 20. Model checks for the rice-total abundance model. a. Plot of fitted values vs. Pearson residuals b. Q-Q plot to check the normality of the residuals. c. Plot of observed vs predicted values.

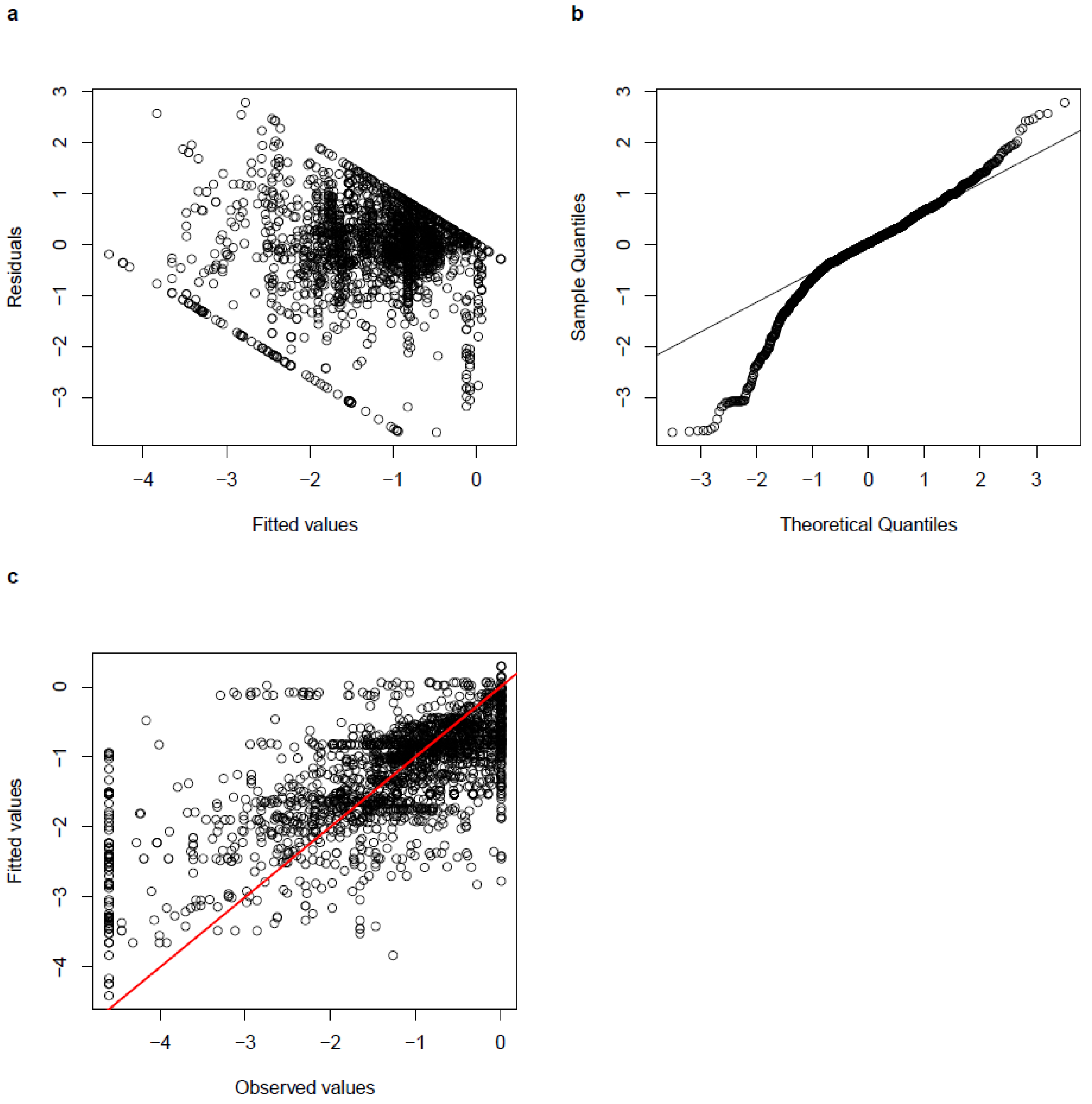

Relative abundance-weighted average range-size (RCAR)

Supplementary Figure 21. Model checks for the land conversion-RCAR model. a. Plot of fitted values vs. Pearson residuals b. Q-Q plot to check the normality of the residuals. c. Plot of observed vs predicted values.

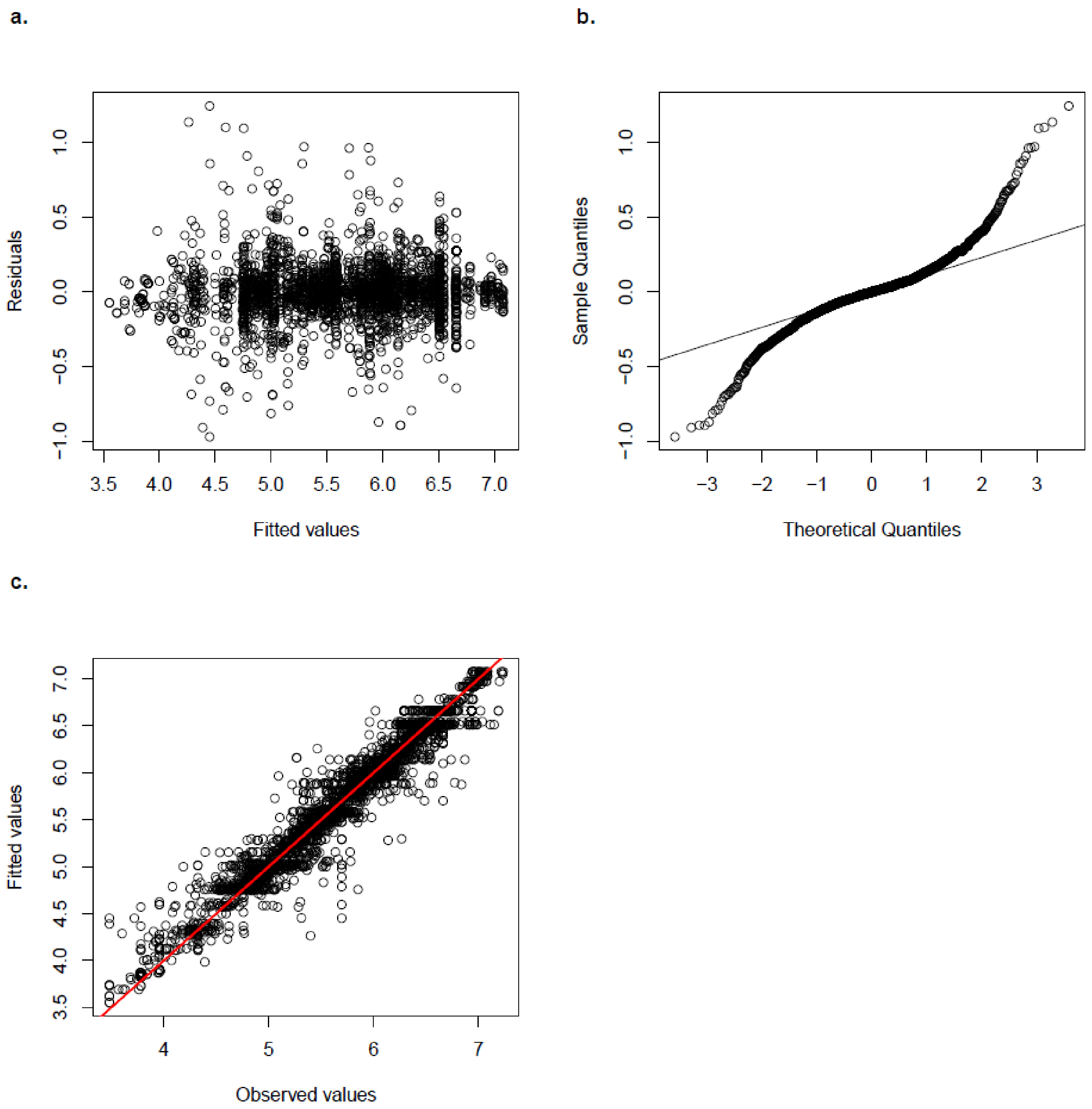

Supplementary Figure 22. Model checks for the maize-RCAR model. a. Plot of fitted values vs. Pearson residuals b. Q-Q plot to check the normality of the residuals. c. Plot of observed vs predicted values.

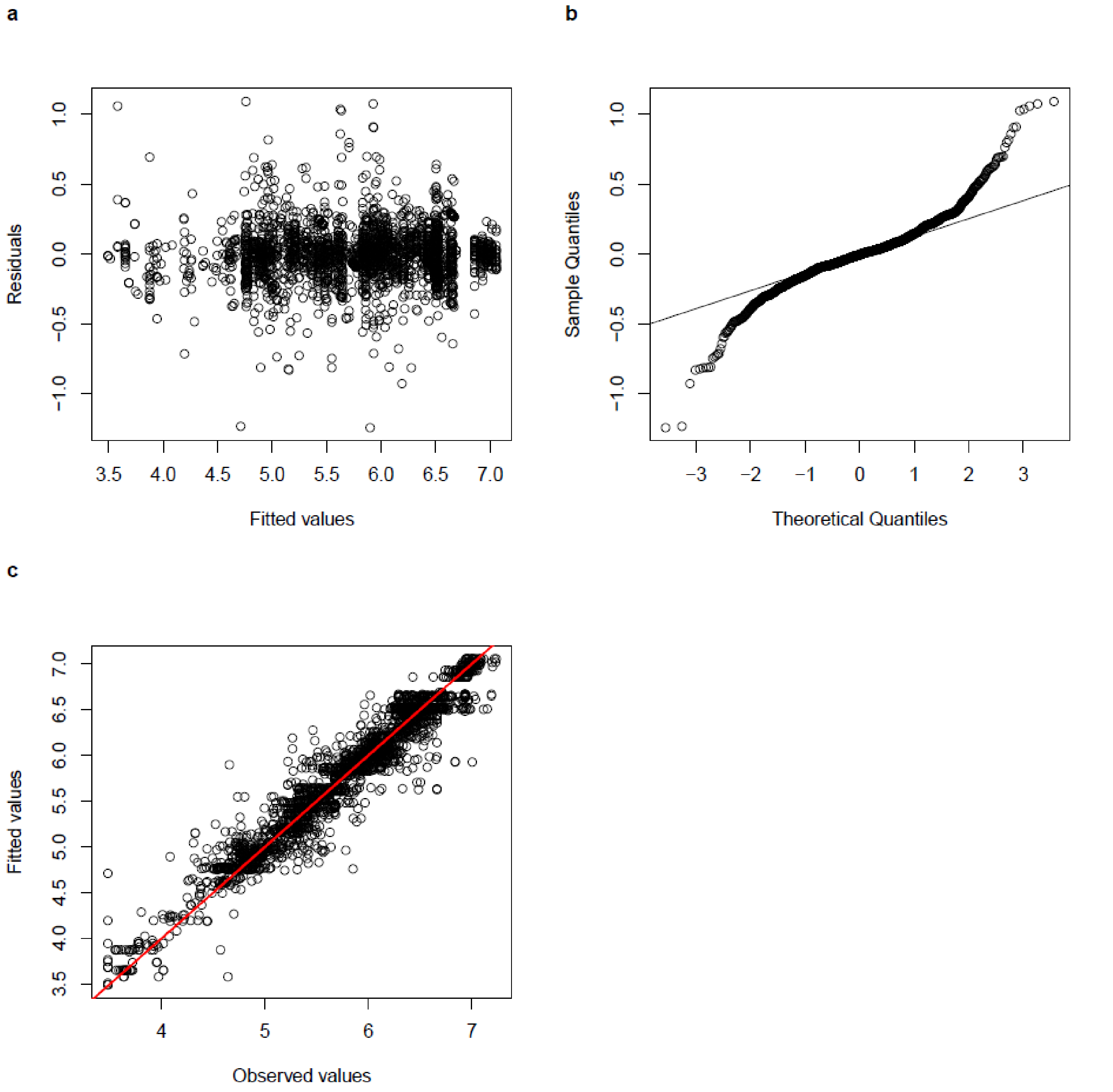

Supplementary Figure 23. Model checks for the soy-RCAR model. a. Plot of fitted values vs. Pearson residuals b. Q-Q plot to check the normality of the residuals. c. Plot of observed vs predicted values.

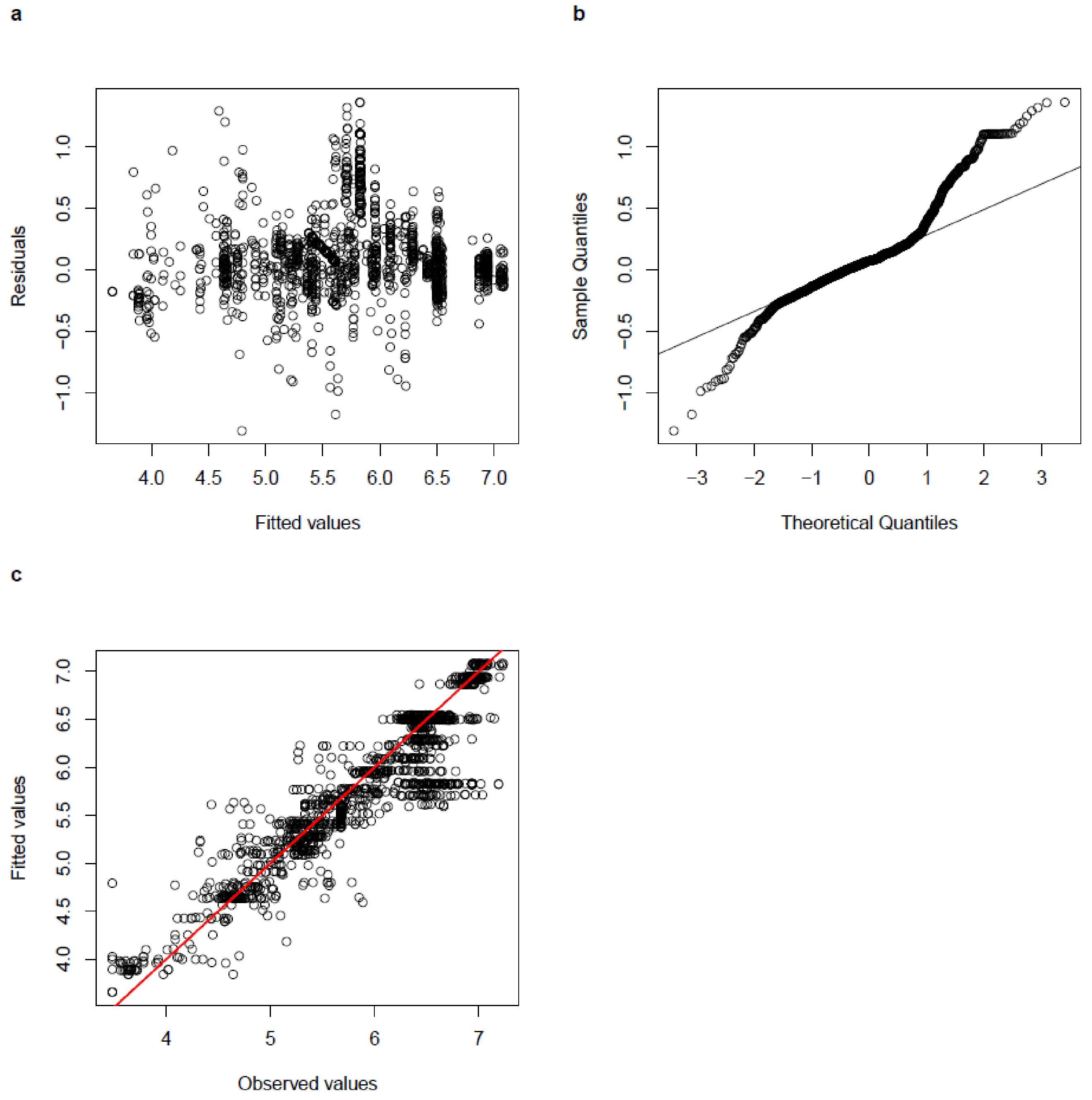

Supplementary Figure 24. Model checks for the wheat-RCAR model. a. Plot of fitted values vs. Pearson residuals b. Q-Q plot to check the normality of the residuals. c. Plot of observed vs predicted values.

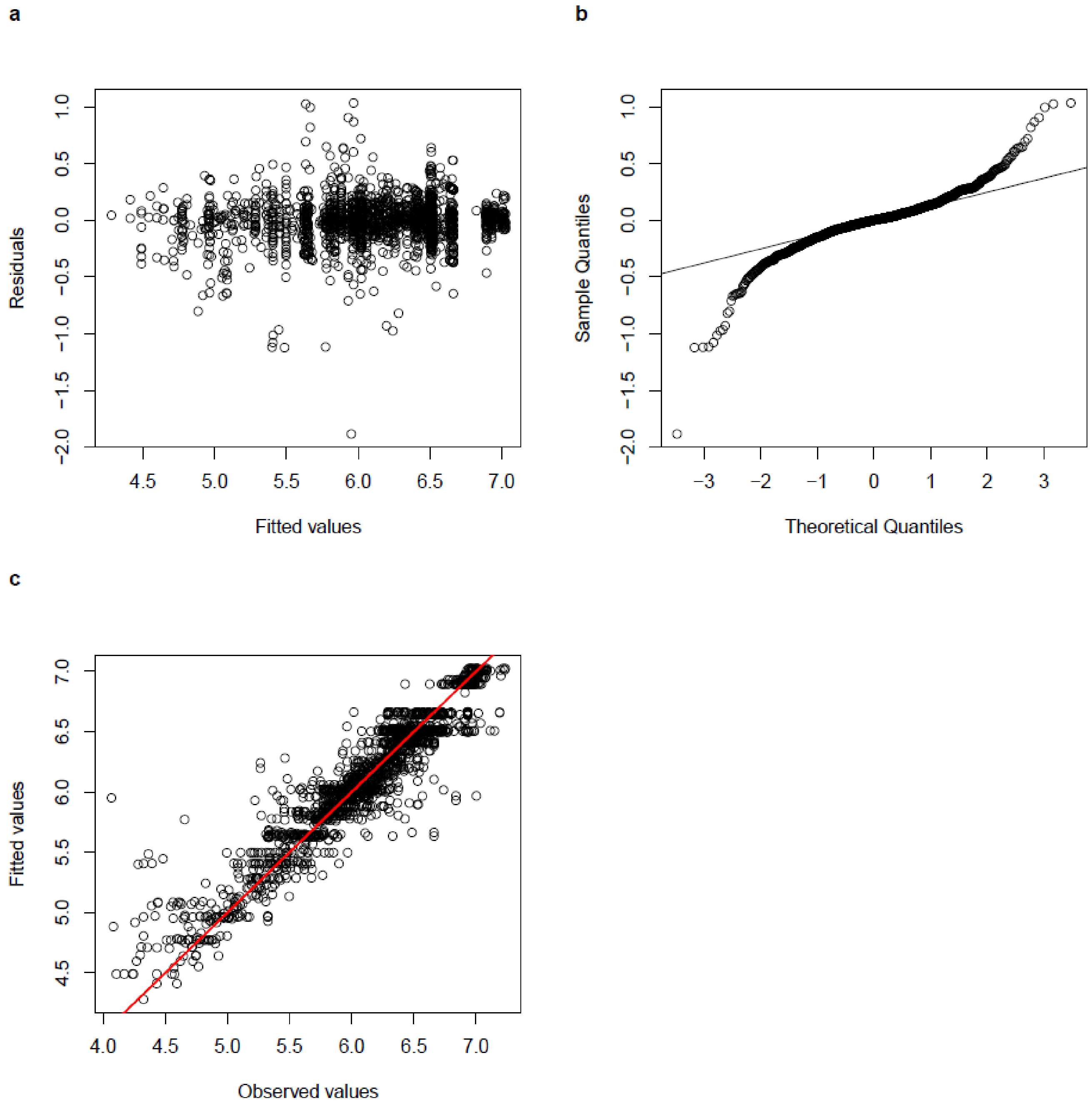

Supplementary Figure 25. Model checks for the rice-RCAR model. a. Plot of fitted values vs. Pearson residuals b. Q-Q plot to check the normality of the residuals. c. Plot of observed vs predicted values.

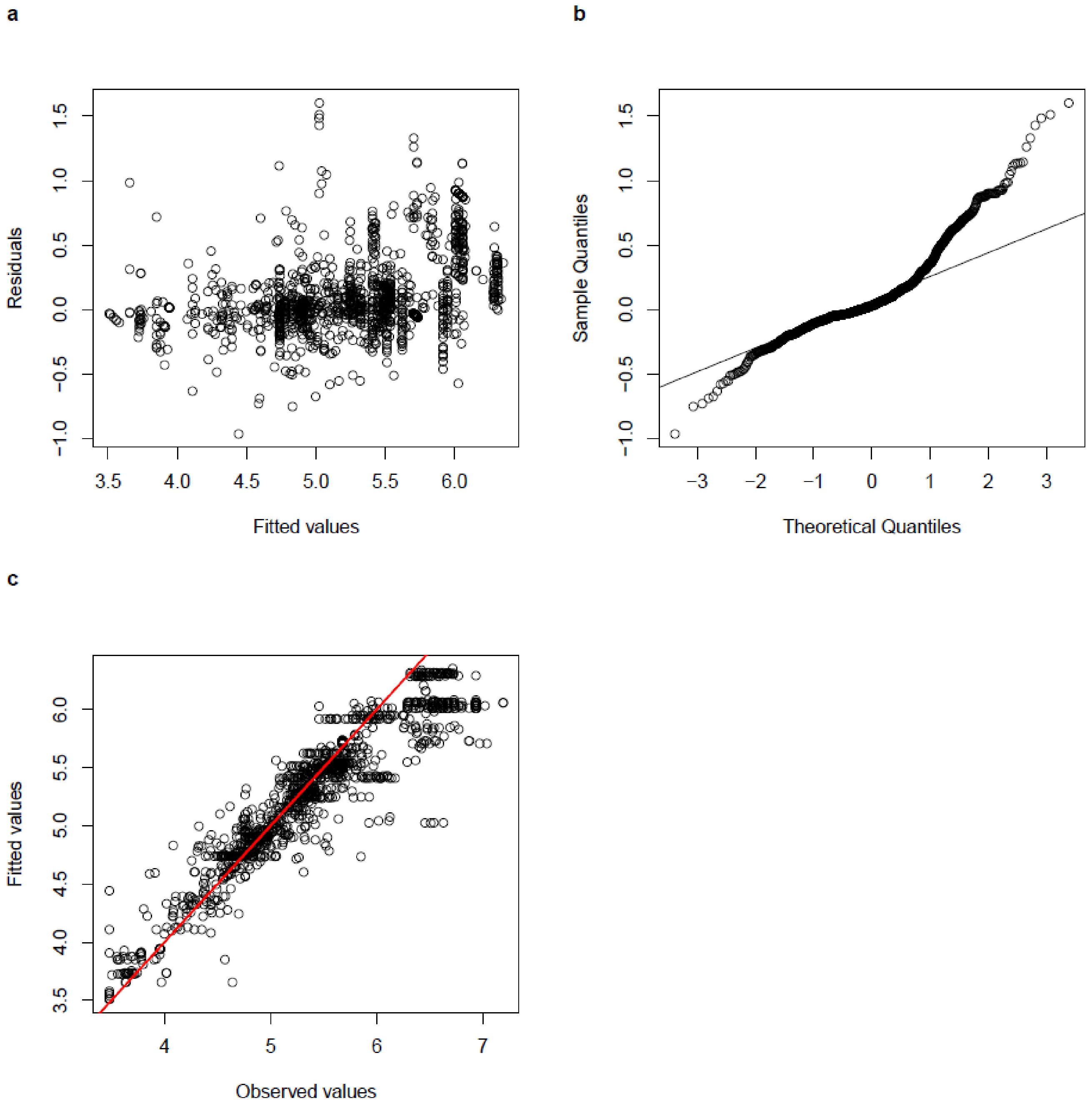

Supplementary Figure 26. The complete set yield effects on three biodiversity metrics, including trends for all geographic regions, land use types and percentage of natural vegetation levels irrespective of the significance of the interactions of these variables with yield. The number of data points for each model can be found in Supplementary Tables 1-5. The plots are organised in rows for each crop: maize (a, e, i); soy (b, f, j); wheat (c, g ,k); rice (d, h, l) and in columns for each biodiversity metric: species richness (a, b, c, d); total abundance (e, f, g, h); relative abundance-weighted community-average range size (RCAR) (I, j, k, l). The plots show the responses of biodiversity metrics to yield, where selected in the final model. If the following variables were also selected in the final model, the different colours and line types have the following meaning: for geographic region – red and blue hues represent tropical and non-tropical regions, respectively; for percentage of natural habitat – dark and light hues represent responses at 85% and 15% natural habitat, respectively; and land use – solid and dashed lines represent cropland and primary vegetation, respectively. If geographic region was not significant either on its own or in interactions, the trend is represented in grey. For cases where yield was not selected in the best model, the plot was left empty. The lines represent median predicted values and the shaded areas represent 95% confidence intervals. Species richness and total abundance increases are usually associated with positive biodiversity changes while RCAR increases are associated with negative biodiversity changes (i.e., a homogenisation of community composition). The red and blue ticks at the bottom of each plot illustrate the yield values in tropical and non-tropical regions, respectively, rescaled to a [0, 1] interval. The plots illustrate model predictions that do not necessarily represent plausible combinations of yield and other variable values. The biodiversity metrics were scaled to the level of the respective metric in primary vegetation in agricultural landscapes with the lowest yield, a value of 0% meaning that the biodiversity level has not changed compared to these reference values. Due to the large range of values, the changes in total abundance (e, f, g, h) and RCAR (I, j, k, l) were $\log_{10}$-transformed to facilitate illustration, and the y-scale is on a logarithmic scale.

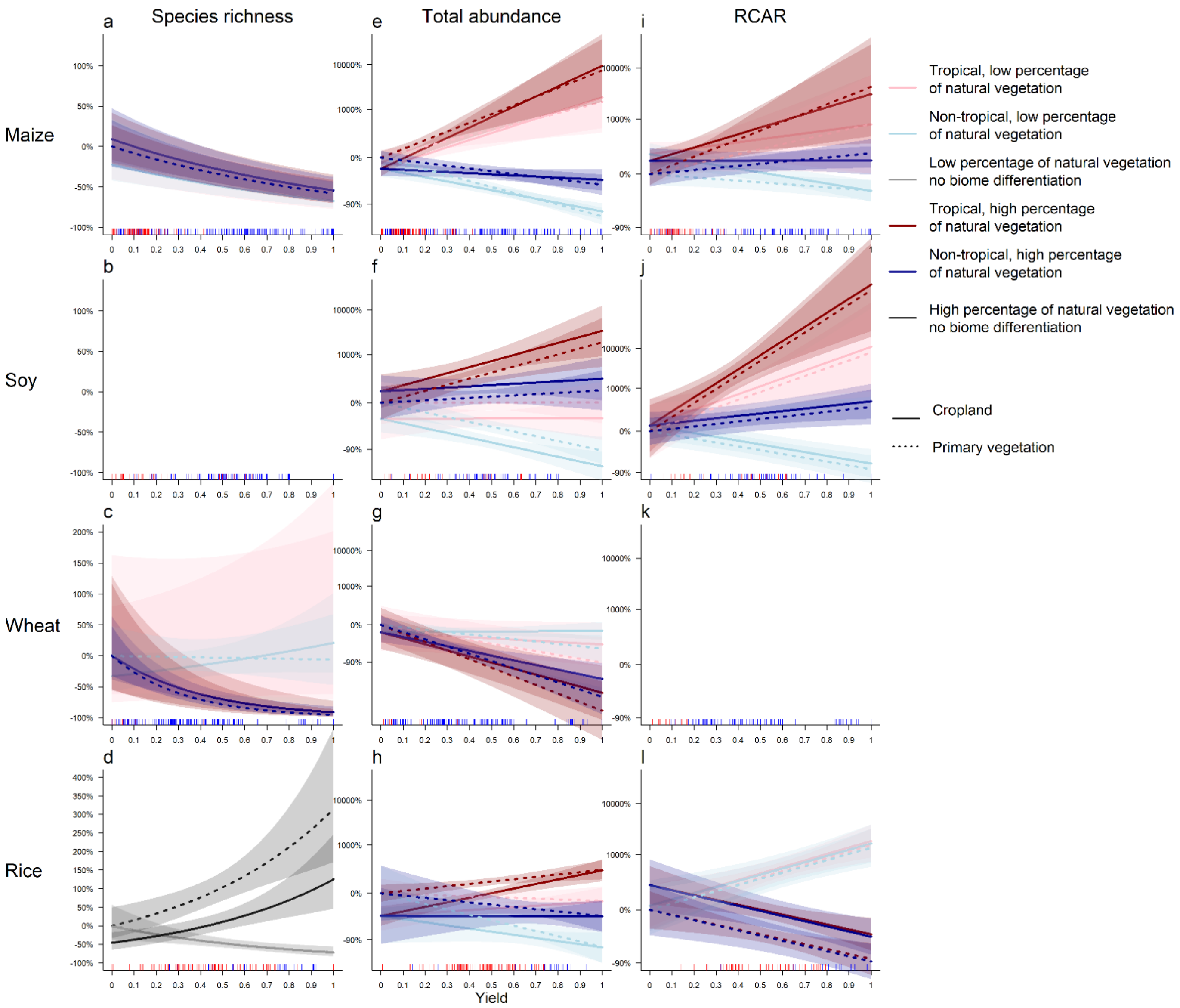

Species richness
Total abundance
RCAR
Maize
Soy
Wheat
Rice
Yield
Tropical, low percentage of natural vegetation
Non-tropical, low percentage of natural vegetation
Low percentage of natural vegetation no biome differentiation
Tropical, high percentage of natural vegetation
Non-tropical, high percentage of natural vegetation
High percentage of natural vegetation no biome differentiation
Cropland
Primary vegetation

Crop and yield gaps spatial data

Supplementary Figure 27. Amount of hectares within each pixel cultivated with a. maize, b. soybean, c. wheat and d. rice. Data available from Monfreda et al[1].

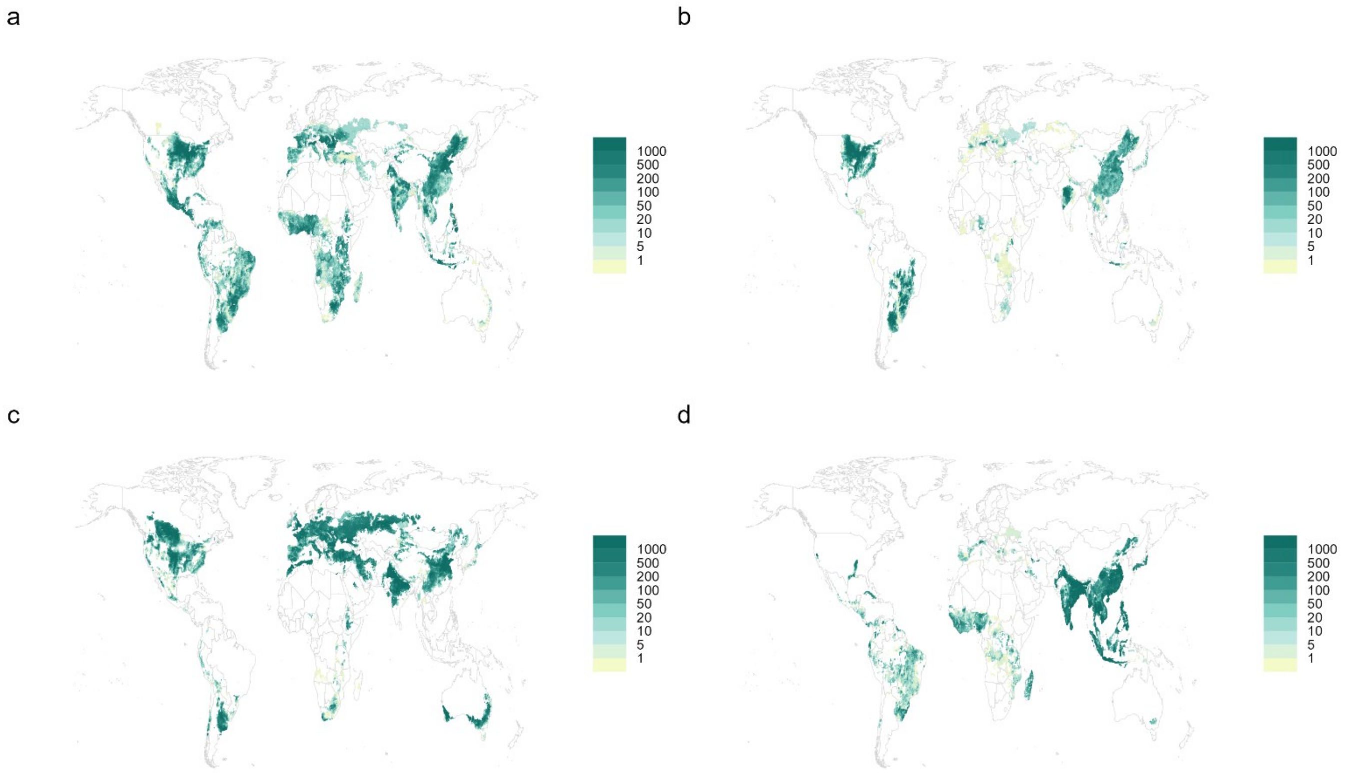

Supplementary Figure 28. Yield gaps estimated by to have existed in 2000 for a. maize, b. soy, c. wheat and d. rice as calculated by Mueller et al[15]. The unit of the scale if tonne/ha.

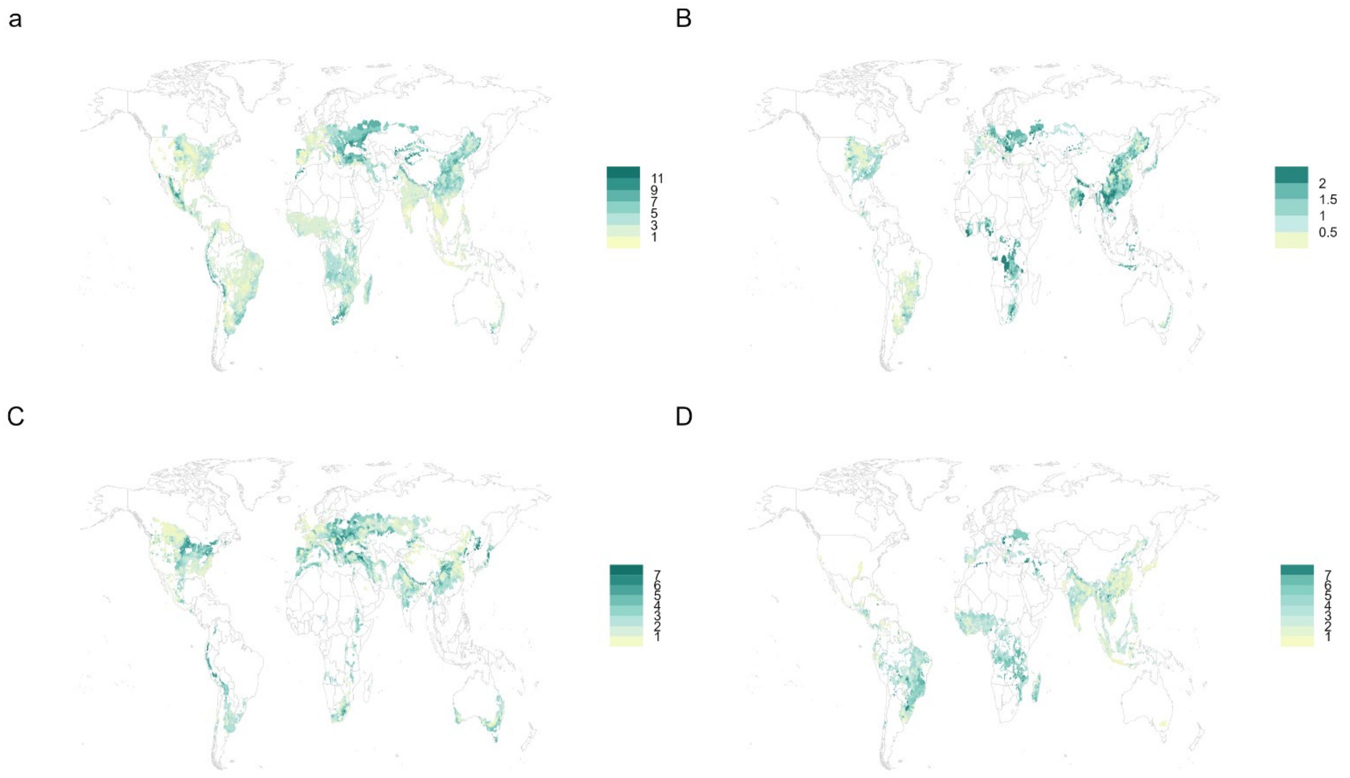

Supplementary Figure 29. The projected effect of closing yield gaps for a. maize, b. soy, c. wheat and d. rice on species richness, calculated as the percentage difference between species richness at yield levels equal to those where yield gaps estimated to have existed in 2000 have been closed, and biodiversity at yield levels estimated for the year 2000.

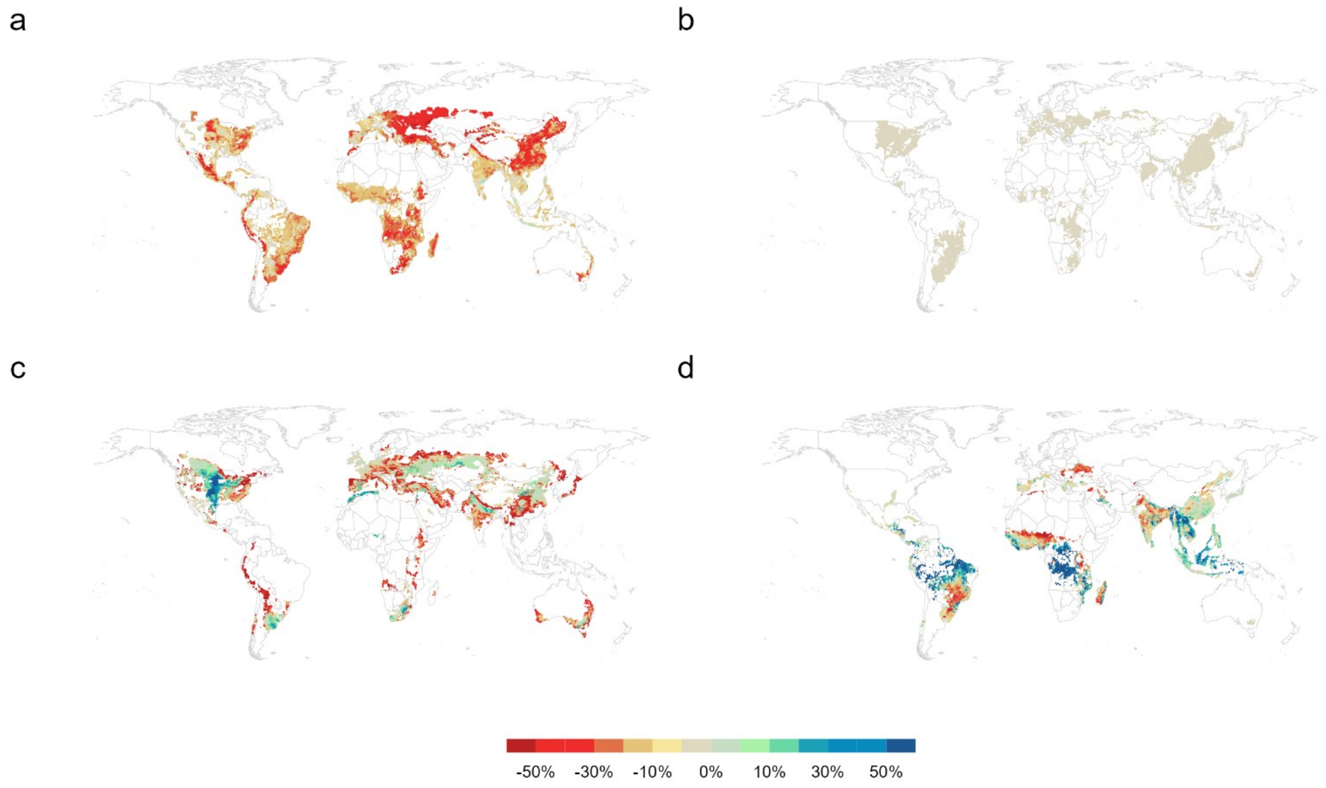

Supplementary Figure 30. The projected effect of closing yield gaps for a. maize, b. soy, c. wheat and d. rice on total abundance, calculated as the percentage difference between abundance at yield levels equal to those where yield gaps estimated to have existed in 2000 have been closed, and abundance at yield levels estimated for the year 2000.

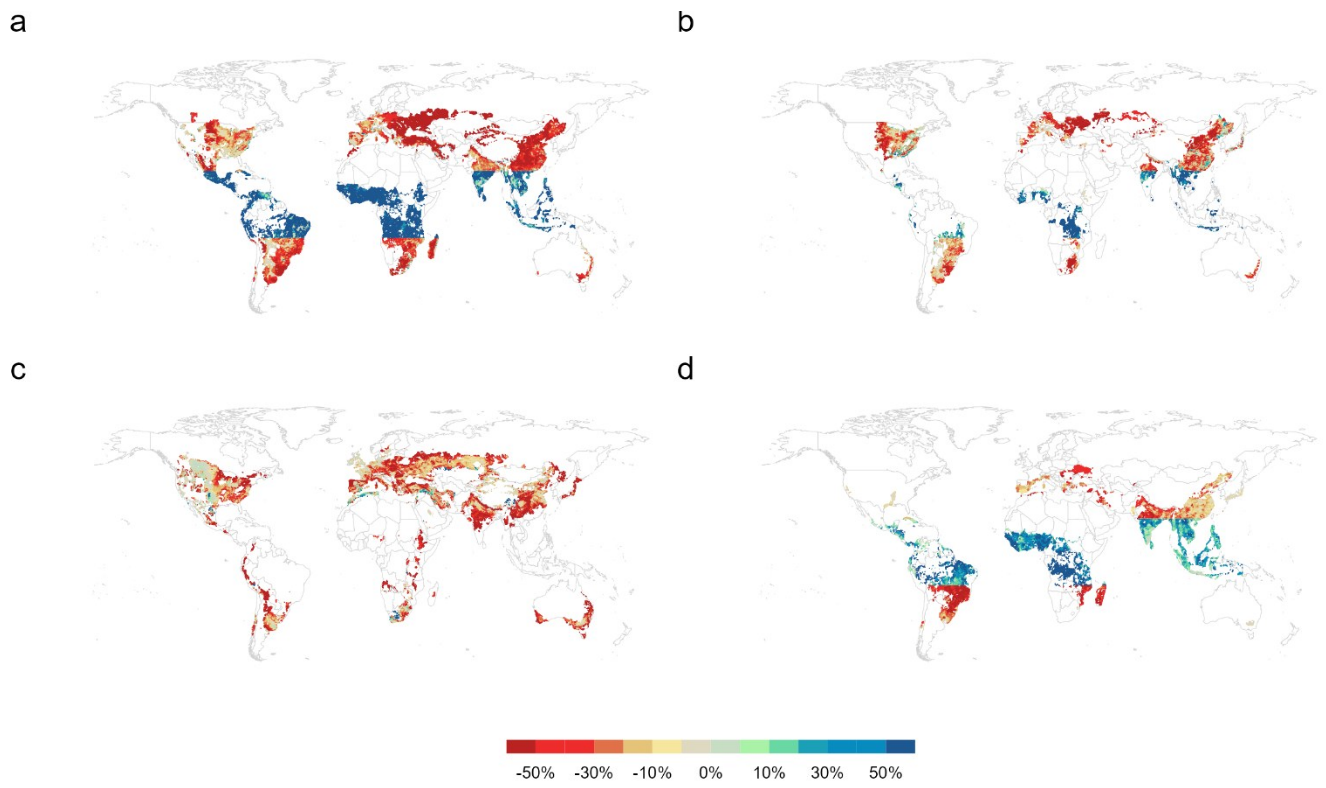

Supplementary Figure 31. The projected effect of closing yield gaps for a. maize, b. soy, c. wheat and d. rice on relative abundance-weighted community-average range size (RCAR), calculated as the percentage difference between RCAR at yield levels equal to those where yield gaps estimated to have existed in 2000 have been closed, and RCAR at yield levels estimated for the year 2000.

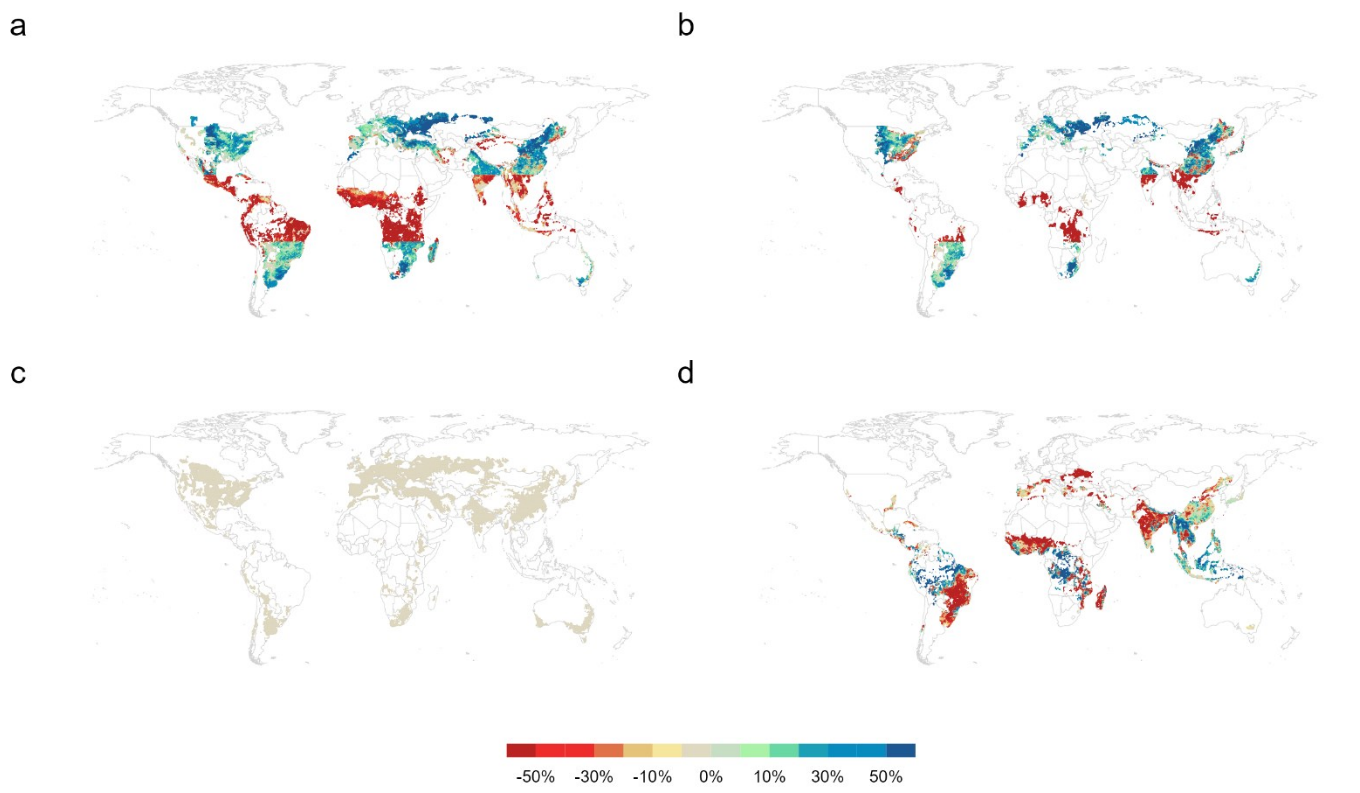

Tests of assumptions of the expansion and intensification scenarios

Supplementary Figure 32. The difference in biodiversity metrics when comparing land expansion and intensification for a 10% production increase. The plots are organised in rows for each crop: maize (a, e, i); soy (b, f, j); wheat (c, g, k); rice (d, h, l) and in columns for each biodiversity metric: species richness (a, b, c, d); total abundance (e, f, g, h); relative abundance-weighted community-average range size (RCAR) (i, j, k, l). The colour range symbolises areas where increasing total production by 10% through cropland expansion is better for biodiversity (blue hues) and areas where increasing total production by 10% through intensification is better for biodiversity (red hues). RCAR (i, j, k, l) increases and decreases were considered to be the negative and positive outcomes for biodiversity, respectively. We removed 5.1%, 9.1%, 4.5% and 1.7% of raster cells from the maize, soybean, wheat and rice analyses, respectively, due to invalid land use coverages (more than 100% cropland coverage or negative coverage of primary vegetation) resulting from the 10% expansion scenario.

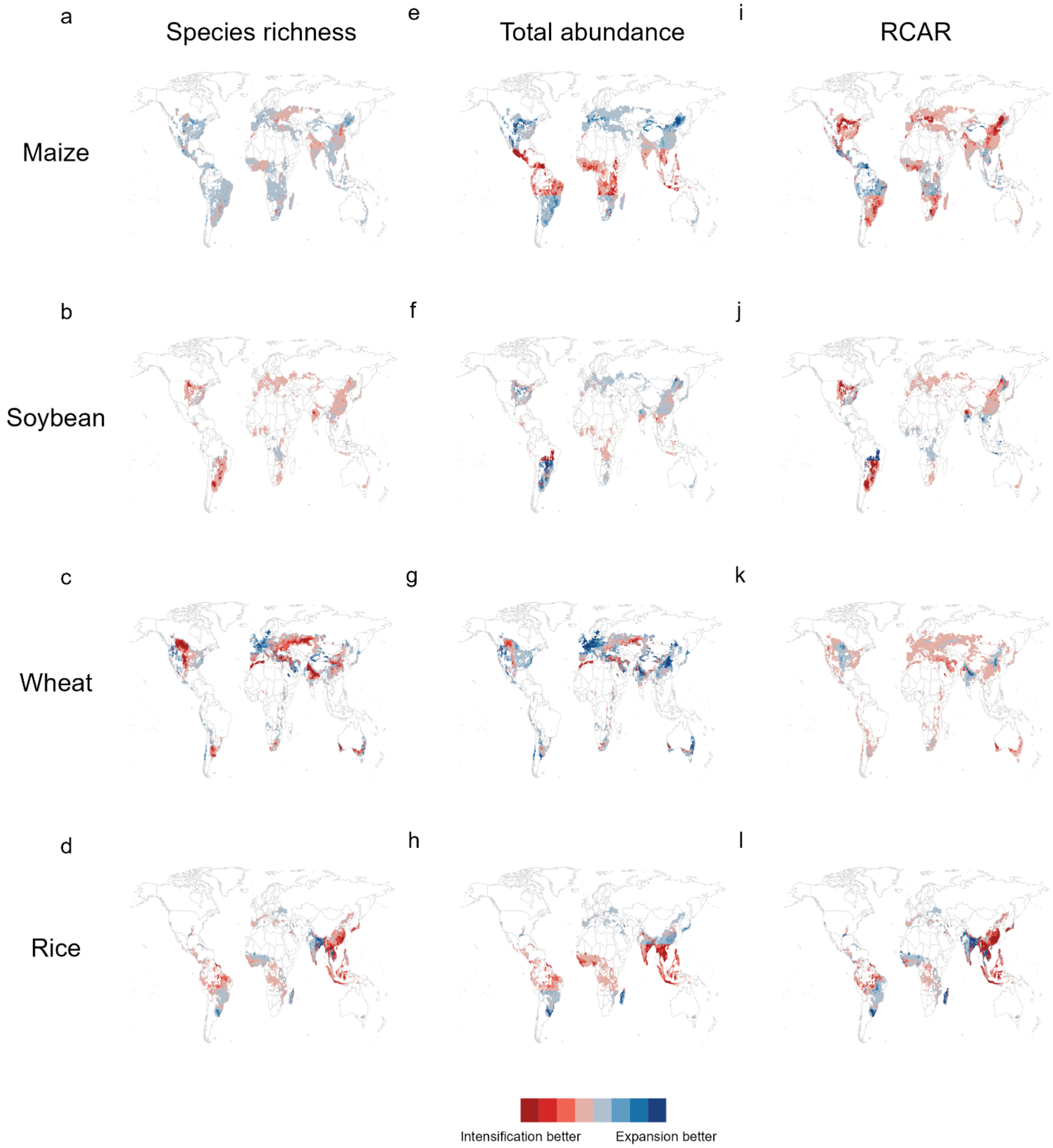

Supplementary Figure 33. The difference in biodiversity metrics when comparing land expansion and intensification on 50% of cropland for a 1% production increase. The plots are organised in rows for each crop: maize (a, e, i); soy (b, f, j); wheat (c, g, k); rice (d, h, l) and in columns for each biodiversity metric: species richness (a, b, c, d); total abundance (e, f, g, h); relative abundance-weighted community-average range size (RCAR) (i, j, k, l). The colour range symbolises areas where increasing total production by 1% through cropland expansion is better for biodiversity (blue hues) and areas where increasing total production by 1% through intensifying 50% of the cropland area for a 2% yield increase is better for biodiversity (red hues). RCAR (i, j, k, l) increases and decreases were considered to be the negative and positive outcomes for biodiversity, respectively. We removed 0.9%, 2.3%, 0.5% and 0.2% of raster cells from the maize, soybean, wheat and rice analyses, respectively, due to invalid land use coverages (more than 100% cropland coverage or negative coverage of primary vegetation) resulting from the 1% expansion scenario.

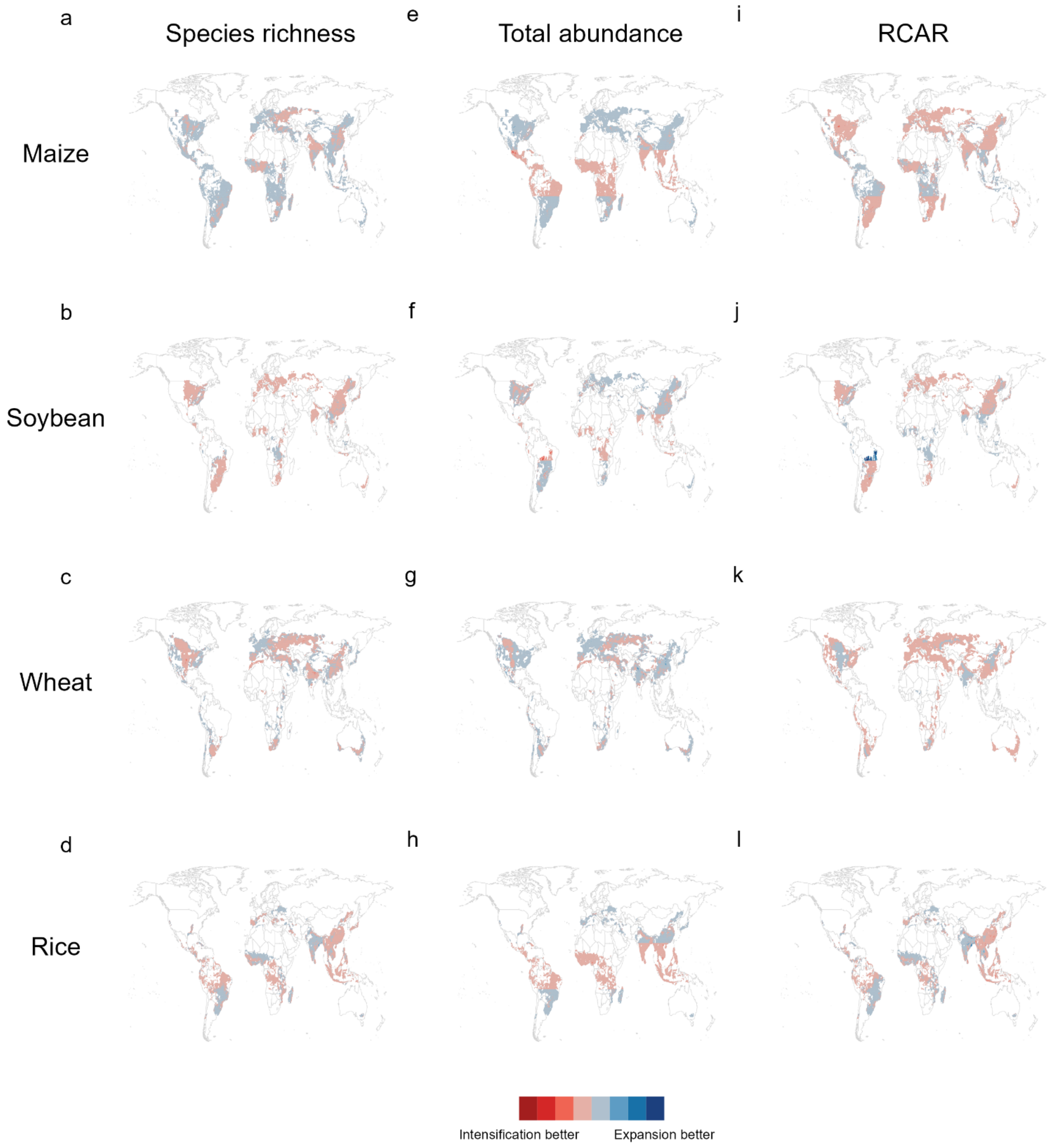

Supplementary Figure 34. The difference in biodiversity metrics when comparing land expansion and intensification on 10% of cropland for a 1% production increase. The plots are organised in rows for each crop: maize (a, e, i); soy (b, f, j); wheat (c, g, k); rice (d, h, l) and in columns for each biodiversity metric: species richness (a, b, c, d); total abundance (e, f, g, h); relative abundance-weighted community-average range size (RCAR) (i, j, k, l). The colour range symbolises areas where increasing total production by 1% through cropland expansion is better for biodiversity (blue hues) and areas where increasing total production by 1% through intensifying 10% of the cropland area for a 10% yield increase is better for biodiversity (red hues). RCAR (i, j, k, l) increases and decreases were considered to be the negative and positive outcomes for biodiversity, respectively. We removed 0.9%, 2.3%, 0.5% and 0.2% of raster cells from the maize, soybean, wheat and rice analyses, respectively, due to invalid land use coverages (more than 100% cropland coverage or negative coverage of primary vegetation) resulting from the 1% expansion scenario.

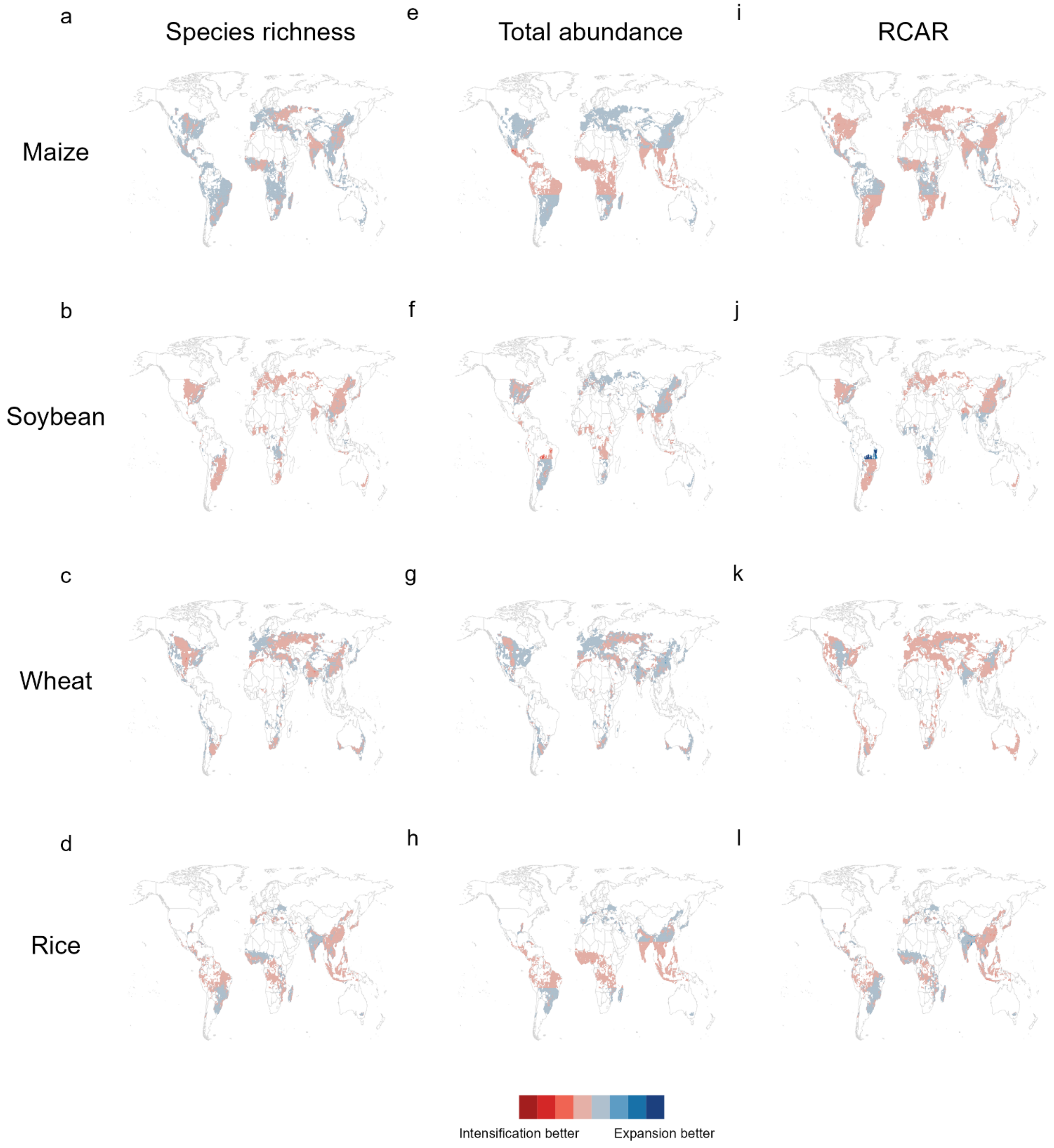

Supplementary Figure 35. The difference in biodiversity metrics when comparing land expansion and intensification for a production increase of 1% of the local yield gap. The plots are organised in rows for each crop: maize (a, e, i); soy (b, f, j); wheat (c, g, k); rice (d, h, l) and in columns for each biodiversity metric: species richness (a, b, c, d); total abundance (e, f, g, h); relative abundance-weighted community-average range size (RCAR) (i, j, k, l). The colour range symbolises areas where increasing total production by 1% of the local yield gap through cropland expansion is better for biodiversity (blue hues) and areas where increasing total production by 1% of the local yield gap through increasing yields is better for biodiversity (red hues). RCAR (i, j, k, l) increases and decreases were considered to be the negative and positive outcomes for biodiversity, respectively. We removed 0.4%, 0.3%, 0.3% and 0.1% of raster cells from the maize, soybean, wheat and rice analyses, respectively, due to invalid land use coverages (more than 100% cropland coverage or negative coverage of primary vegetation) resulting from the 1% expansion scenario.

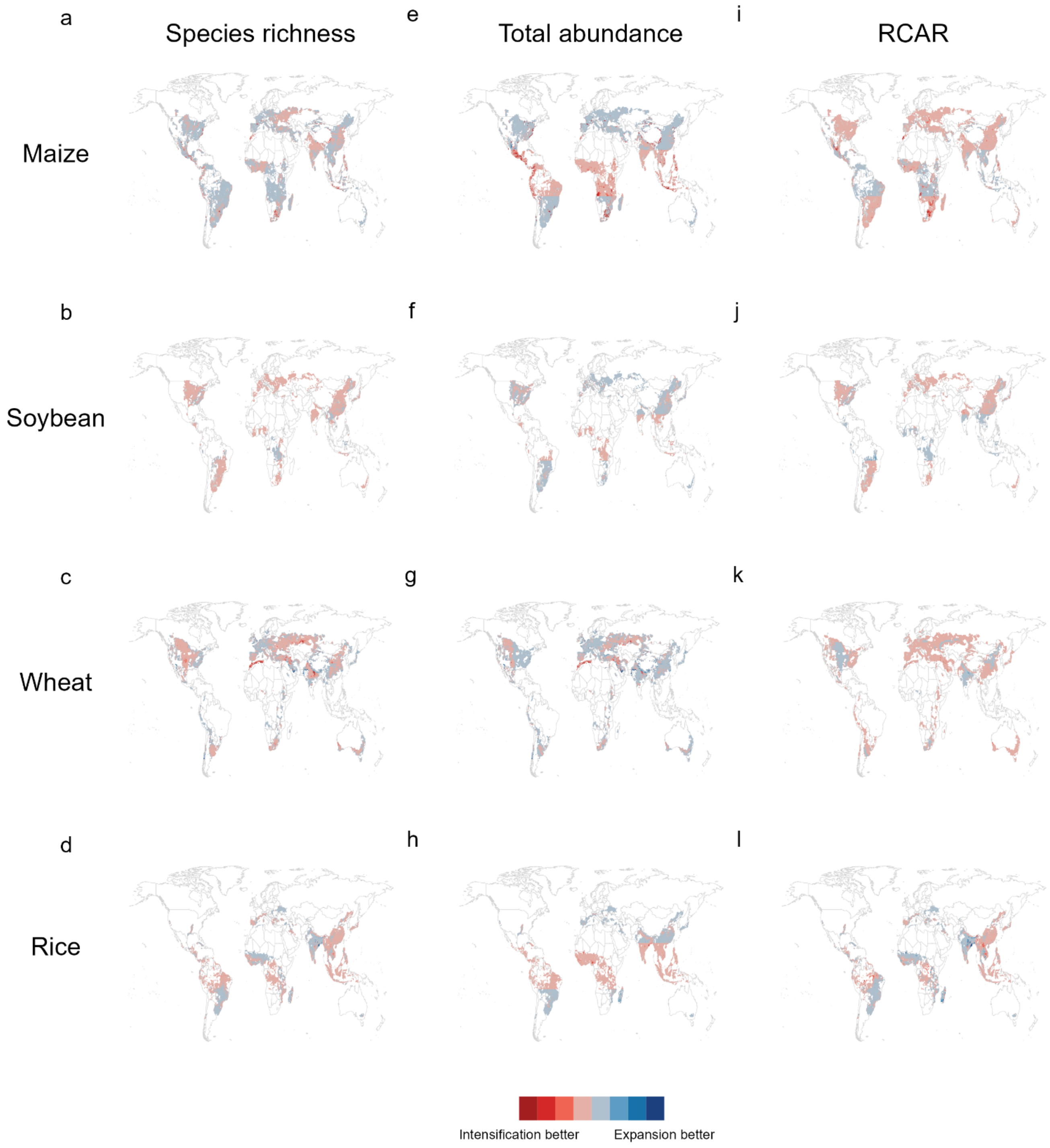

Supplementary Figure 36. The difference in biodiversity metrics when comparing land expansion and intensification + restoration for a 1% production increase. The plots are organised in rows for each crop: maize (a, e, i); soy (b, f, j); wheat (c, g, k); rice (d, h, l) and in columns for each biodiversity metric: species richness (a, b, c, d); total abundance (e, f, g, h); relative abundance-weighted community-average range size (RCAR) (i, j, k, l). The colour range symbolises areas where increasing total production by 1% through cropland expansion is better for biodiversity (blue hues) and areas where increasing total production by 1% through intensification (10% yield increase) on 91.8% of cropland and restoration on the remaining 8.2% of cropland is better for biodiversity (red hues). RCAR (i, j, k, l) increases and decreases were considered to be the negative and positive outcomes for biodiversity, respectively. We removed 0.9%, 2.3%, 0.5% and 0.2% of raster cells from the maize, soybean, wheat and rice analyses, respectively, due to invalid land use coverages (negative or over 100% coverage for a single land use type) resulting from the 1% expansion scenario. Please note that restored cropland has been converted to primary vegetation in this exercise, despite it not fitting the definition from the main text, in order for our models to be able to project these results.

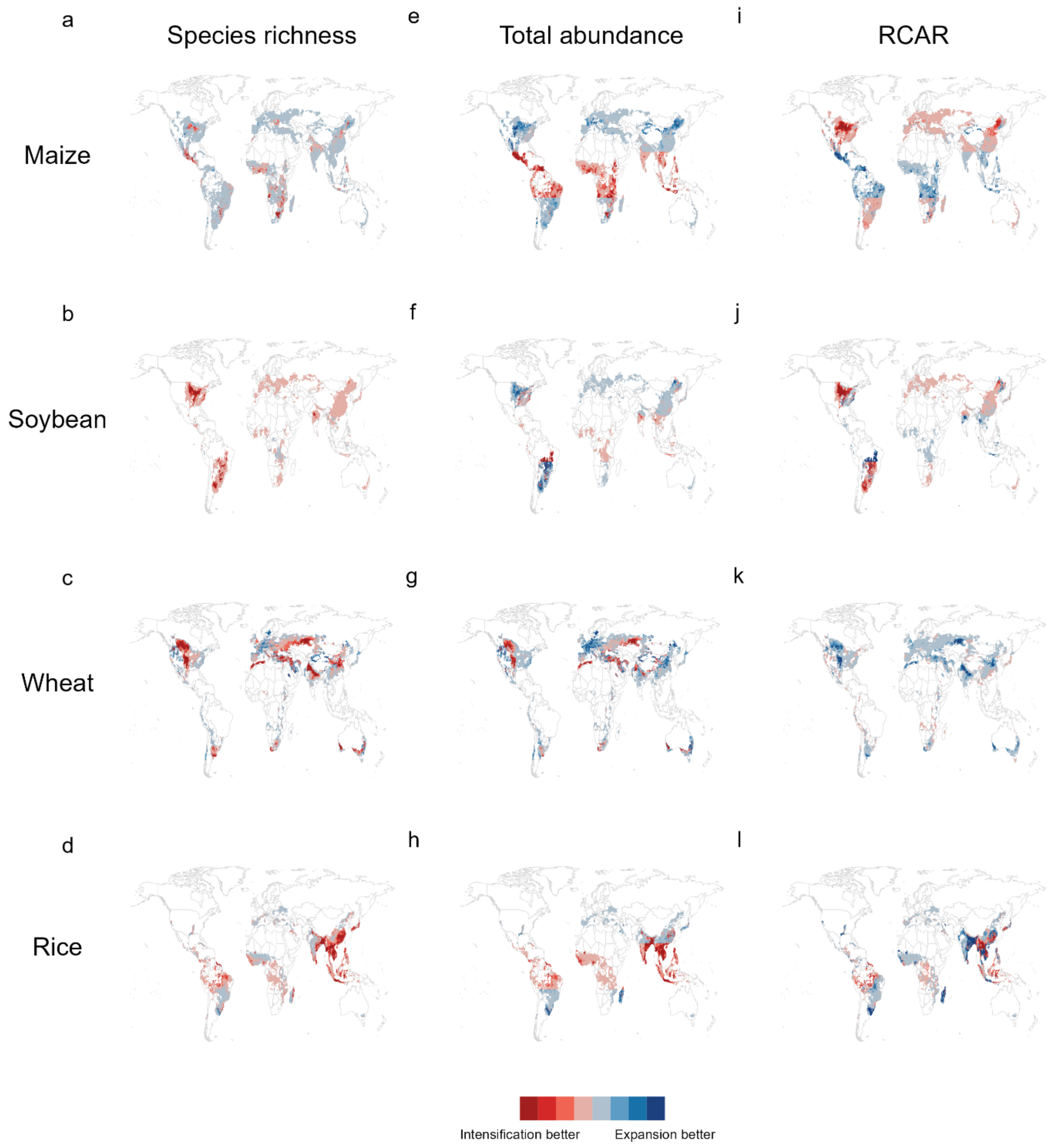

Supplement: Supplementary file 1 — Supplementary Methods, Tables 1–21 and Figs. 1–36. [file 41559_2025_2691_MOESM1_ESM.pdf]
