# Supplementary material for: Geography and availability of natural habitat determine whether cropland intensification or expansion is more detrimental to biodiversity
Source: Nat Ecol Evol. 2025 May 1;9(6):993–1008. doi: 10.1038/s41559-025-02691-x (PMC12148938; doi:10.1038/s41559-025-02691-x)
Supplement: Supplementary file 2 — Reporting Summary [file 41559_2025_2691_MOESM2_ESM.pdf]

nature portfolio

Corresponding author(s): Silvia Ceausu

Last updated by author(s): Jan 9, 2025

Reporting Summary

Nature Portfolio wishes to improve the reproducibility of the work that we publish. This form provides structure for consistency and transparency in reporting. For further information on Nature Portfolio policies, see our Editorial Policies and the Editorial Policy Checklist.

Statistics

For all statistical analyses, confirm that the following items are present in the figure legend, table legend, main text, or Methods section.

n/a	Confirmed	
☐	☒	The exact sample size (*n*) for each experimental group/condition, given as a discrete number and unit of measurement
☐	☒	A statement on whether measurements were taken from distinct samples or whether the same sample was measured repeatedly
☐	☒	The statistical test(s) used AND whether they are one- or two-sided<br>*Only common tests should be described solely by name; describe more complex techniques in the Methods section.*
☐	☒	A description of all covariates tested
☐	☒	A description of any assumptions or corrections, such as tests of normality and adjustment for multiple comparisons
☐	☒	A full description of the statistical parameters including central tendency (e.g. means) or other basic estimates (e.g. regression coefficient) AND variation (e.g. standard deviation) or associated estimates of uncertainty (e.g. confidence intervals)
☐	☒	For null hypothesis testing, the test statistic (e.g. *F*, *t*, *r*) with confidence intervals, effect sizes, degrees of freedom and *P* value noted<br>*Give P values as exact values whenever suitable.*
☐	☒	For Bayesian analysis, information on the choice of priors and Markov chain Monte Carlo settings
☐	☒	For hierarchical and complex designs, identification of the appropriate level for tests and full reporting of outcomes
☐	☒	Estimates of effect sizes (e.g. Cohen's *d*, Pearson's *r*), indicating how they were calculated

Our web collection on statistics for biologists contains articles on many of the points above.

Software and code

Policy information about availability of computer code

Data collection	This study uses existing datasets. No software was used to collect the data specifically for this study.
Data analysis	R 3.6.3 , Packages: glmmTMB version 1.1.7 , DHARMa 0.4.6, brms 2.21.0, raster 3.6-23, StatisticalModels 0.1, predictsFunctions 1.0. The code required to run the analyses presented here can be downloaded from: https://github.com/SilviaCeausu/BiodivYield.

For manuscripts utilizing custom algorithms or software that are central to the research but not yet described in published literature, software must be made available to editors and reviewers. We strongly encourage code deposition in a community repository (e.g. GitHub). See the Nature Portfolio guidelines for submitting code & software for further information.

Data

Policy information about availability of data

All manuscripts must include a data availability statement. This statement should provide the following information, where applicable:

- Accession codes, unique identifiers, or web links for publicly available datasets
- A description of any restrictions on data availability
- For clinical datasets or third party data, please ensure that the statement adheres to our policy

The PREDICTS database used for this study is available from https://data.nhm.ac.uk/dataset/the-2016-release-of-the-predicts-database. PREDICTS site-level biodiversity data with estimates of community-average range size is available from https://figshare.com/articles/dataset/PREDICTS_site-level_biodiversity_data_with_estimates_of_community-average_range_size/7262732. The EarthStat data is available from http://www.earthstat.org/. The

MapSPAM data is available from https://mapspam.info/. The EPIC-BOKU subsistence yield data is publicly available and can be downloaded from: https://figshare.com/articles/dataset/Subsistence_yields_for_maize_soybean_wheat_and_rice_used_for_analysis_in_the_study_Geography_and_availability_of_natural_habitat_determine_whether_cropland_intensification_or_expansion_is_more_detrimental_to_biodiversity_/25780953. The land-use data based on which we calculated the percentage of natural habitat can be downloaded from http://doi.org/10.4225/08/56DCD9249B224. HYDE database underlying the duration of substantial human modification data can be downloaded at: https://landuse.sites.uu.nl/datasets/. The climate variables can be downloaded from https://www.worldclim.org/data/index.html.

Source data for figure 4 can be downloaded from: https://figshare.com/articles/journal_contribution/Figure_4_The_projected_effect_of_closing_yield_gaps_on_three_biodiversity_metrics_a_local_species_richness_b_total_abundance_and_c_relative_abundance-weighted_community-average_range_size_RCAR_/28592318?file=52978718.

Source data for figure 5 can be downloaded from: https://figshare.com/articles/journal_contribution/Figure_5_The_difference_in_biodiversity_metrics_when_comparing_land_expansion_and_intensification_within_the_same_agricultural_landscape_/28592387?file=52979207.

The data tables obtained from extracting information at the location of PREDICTS sites and used in the the statistical analyses can be downloaded from: https://figshare.com/articles/dataset/Datasets_used_for_modelling_the_impact_of_land_conversion_yield_on_biodiversity_for_the_article_Geography_and_availability_of_natural_habitat_determine_whether_cropland_intensification_or_expansion_is_more_detrimental_to_biodiversity_/28592393.

Research involving human participants, their data, or biological material

Policy information about studies with human participants or human data. See also policy information about sex, gender (identity/presentation), and sexual orientation and race, ethnicity and racism.

Reporting on sex and gender	NA
Reporting on race, ethnicity, or other socially relevant groupings	NA
Population characteristics	NA
Recruitment	NA
Ethics oversight	NA

Note that full information on the approval of the study protocol must also be provided in the manuscript.

Field-specific reporting

Please select the one below that is the best fit for your research. If you are not sure, read the appropriate sections before making your selection.

☐ Life sciences ☐ Behavioural & social sciences ☒ Ecological, evolutionary & environmental sciences

For a reference copy of the document with all sections, see nature.com/documents/nr-reporting-summary-flat.pdf

Ecological, evolutionary & environmental sciences study design

All studies must disclose on these points even when the disclosure is negative.

Study description

This study provides a quantitative analysis of biodiversity effects to land conversion and yield increases, including closing yield gaps. The land conversion (independent of yield increases) analysis focused on cropland and primary vegetation in either largely unmodified landscapes or in highly modified landscapes. The yield increases analysis (accounting for amount of natural habitat) focuses on 4 crops: maize, soy, wheat and rice. We also use projections of the biodiversity effects models to compare farmland expansion and intensification in agricultural landscapes.
We use a subset of 1,318,867 cropland and primary vegetation records from 10,094 sites of the freely available PREDICTS database alongside publicly available yield and land-use data. These records were further selected based on landscape composition for the land conversion models (5328 sites), and to match the extent of maize (4862 sites), soy (2404 sites), wheat (3227 sites) and rice (2810 sites) cultivation extent according to the publicly available EarthStat data, which we used for yield and yield gap information. The sites in the biodiversity data are nested within spatial blocks, which are nested within studies. We build mixed effects models for three biodiversity metrics: sampled species richness, total sampled relative abundance, and relative abundance-weighted community-average range size (RCAR). The models included interactions between yield and natural habitat, and interactions of each of these two with subsistence yield, land use and biome.

Research sample

We used the 2016 release of the PREDICTS database, which contains 3,250,404 biodiversity records, mostly sampled from 2000 to 2012, from 666 published studies. Each study within the PREDICTS database contains data sampled with the same method across a gradient of land use or land-use intensity. The data in each study are grouped into one or more spatial blocks, each containing data from one or more sites. Each site is attributed one of 6 predominant land-use classes based on the information provided in the original papers, or by the authors of those papers. The subset of the data used in this study includes 18,853 species of which 3,994

are vertebrates, 6,693 are invertebrates, 7,269 are plants, 894 are fungi, and three are protists.
The PREDICTS database used for this study is available from https://data.nhm.ac.uk/dataset/the-2016-release-of-the-predicts-database. PREDICTS site-level biodiversity data with estimates of community-average range size is available from https://figshare.com/articles/dataset/PREDICTS_site-level_biodiversity_data_with_estimates_of_community-average_range_size/7262732.
The EarthStat data is available from http://www.earthstat.org/. The land-use data based on which we calculated the percentage of natural habitat can be downloaded from http://doi.org/10.4225/08/56DCD9249B224.

Sampling strategy	For the land conversion analysis, we used all PREDICTS cropland and primary vegetation sites that were in landscapes with either more than 70% natural habitat (unmodified landscape) or in landscape with less than 30% natural habitat (modified landscape). For the yield analysis, we used all sites that had relatively high quality information on yield based on EarthStat data for maize, soy, wheat and rice.
Data collection	All the datasets used for this analysis were either collected or modelled as described in the methods of this article. No new data were collected for this study.
Timing and spatial scale	Most biodiversity data were collected from 2000 to 2012. The frequency and periodicity differs for each study included in the PREDICTS database. EarthStat data represent yield, area and yield gap estimates for the year 2000, which are based on information for the years between 1997 and 2003. The land-use data that we used to estimate amount of natural habitat are estimates for the year 2005. Both the EarthStat and land-use data years are within the sampling timespan for the PREDICTS data.
Data exclusions	We only excluded data points that intersected with EarthStat yield estimates that were based on lower quality information as described in the methods section. This exclusion criteria was pre-established.
Reproducibility	All datasets and code are freely available.
Randomization	All data has been collected and published before this study. Therefore, randomization was not possible. Differences due to study design were accounted for by the use random effects. Covariates were controlled for by inclusion in the statistical models.
Blinding	Blinding was not possible for this analysis because all the data were collected before the start of this study.

Did the study involve field work? ☐ Yes ☒ No

Reporting for specific materials, systems and methods

We require information from authors about some types of materials, experimental systems and methods used in many studies. Here, indicate whether each material, system or method listed is relevant to your study. If you are not sure if a list item applies to your research, read the appropriate section before selecting a response.

Materials & experimental systems

n/a	Involved in the study
☒	☐ Antibodies
☒	☐ Eukaryotic cell lines
☒	☐ Palaeontology and archaeology
☒	☐ Animals and other organisms
☒	☐ Clinical data
☒	☐ Dual use research of concern
☒	☐ Plants

Methods

n/a	Involved in the study
☒	☐ ChIP-seq
☒	☐ Flow cytometry
☒	☐ MRI-based neuroimaging

Plants

Seed stocks	*Report on the source of all seed stocks or other plant material used. If applicable, state the seed stock centre and catalogue number. If plant specimens were collected from the field, describe the collection location, date and sampling procedures.*
Novel plant genotypes	*Describe the methods by which all novel plant genotypes were produced. This includes those generated by transgenic approaches, gene editing, chemical/radiation-based mutagenesis and hybridization. For transgenic lines, describe the transformation method, the number of independent lines analyzed and the generation upon which experiments were performed. For gene-edited lines, describe the editor used, the endogenous sequence targeted for editing, the targeting guide RNA sequence (if applicable) and how the editor was applied.*
Authentication	*Describe any authentication procedures for each seed stock used or novel genotype generated. Describe any experiments used to assess the effect of a mutation and, where applicable, how potential secondary effects (e.g. second site T-DNA insertions, mosiacism, off-target gene editing) were examined.*
